# Genome-wide analysis of sugar transporter gene family in *Erianthus rufipilus* and *Saccharum officinarum*, expression profiling and identification of transcription factors

**DOI:** 10.3389/fpls.2024.1502649

**Published:** 2025-01-09

**Authors:** Sehrish Akbar, Xuiting Hua, Yingying Zhang, Gang Liu, Tianyou Wang, Huihong Shi, Zhen Li, Yiying Qi, Habiba Habiba, Wei Yao, Mu-Qing Zhang, Jisen Zhang

**Affiliations:** ^1^ Center for Genomics and Biotechnology, National Sugarcane Engineering Technology Research Center, Fujian Provincial Key Laboratory of Haixia Applied Plant Systems Biology, College of Agriculture, Fujian Agriculture and Forestry University, Fuzhou, China; ^2^ State Key Laboratory for Conservation and Utilization of Subtropical Agro-bioresources, Guangxi Key Laboratory of Sugarcane Biology, Guangxi University, Guangxi, China; ^3^ Department of Biological Science, Lehman College, City University of New York, Bronx, NY, United States

**Keywords:** sugar transporter, genome wide analysis, *Erianthus rufipilus*, gene family analysis, *Saccharum officinarum*, yeast-1-hybrid (Y1H) assay

## Abstract

Sugar, the primary product of photosynthesis, is a vital requirement for cell activities. Allocation of sugar from source to sink tissues is facilitated by sugar transporters (ST). These STs belong to the Major Facilitator Superfamily (MFS), the largest family of STs in plants. In this study, we performed genome wide and gene expression data analysis to identify the putative ST genes in *Erianthus rufipilus (E. rufipilus)* and in *Saccharum officinarum (S. officinarum)*. We identified 78 ST gene families in *E. rufipilus* and 86 ST gene families in *S. officinarum*. Phylogenetic analysis distributed the ST genes into eight distinct subfamilies (INT, MST, VGT, pGlcT, PLT, STP, SFP and SUT). Chromosomal distribution of ST genes clustered them on 10 respective chromosomes. Furthermore, synteny analysis with *S. spontaneum* and *Sorghum bicolor (S. bicolor)* revealed highly colinear regions. Synonymous and non-synonymous ratio (Ka/Ks) showed purifying selection in gene evolution. Promoter analysis identified several *cis*-regulatory elements, mainly associated with light responsiveness. We also examined the expression pattern of ST genes in different developing tissues (mature leaf, pre-mature stem, mature stem and seedling stem). Under sugar stress, we identified the significant ST genes showing differential expression patterns. Moreover, our yeast one-hybrid (Y1H) assays identified NAM, ATAF and CUC (NAC) and Lesion Simulating Disease (LSD) potential transcription factors (TFs) that may bind to the SUT1-T1 promoter in *S. officinarum*, showing negative correlation pattern with SUT1-T1. Our results deepen our understanding of ST gene evolution in *Saccharum* species and will facilitate the future investigation of functional analysis of the ST gene family.

## Introduction

Sugarcane, from the genus *Saccharum* within the tribe Andropogoneae, stands as the world’s leading crop, thriving in both tropical and temperate zones (D’Hont, 2005; Mulyono, 2016). The genus encompasses around 90 genera and about 1270 species, showcasing a wide geographical spread (Kellogg, 2015). Among these, six species (*Saccharum robustum*, *S. spontaneum*, *S. officinarum*, *Saccharum sinense*, *Saccharum barberi*, and *Saccharum edule*) are recognized within the *Saccharum* L. genus, which is further categorized into two major clades: *Saccharum* S. str. and *Erianthus*, with the former including only Old-World taxa (Mukherjee, 1957; Daniels et al., 1987; Besse et al., 1997, 1998).

*S. officinarum*, evolving from *S. robustum*, is noted for its high sugar content and substantial biomass, characterized by a basic chromosome number of 2n = 8x = 80 (Lu et al., 1994; D’Hont et al., 1996; Schenck et al., 2004). Historically, the *Saccharum* genus was considered to include only polyploid species (Wang et al., 2023). However, *E. rufipilus*, a diploid species within the genus, demonstrates significant cold, drought, and disease resistance, making it valuable for sugarcane breeding through interspecific hybridization (Wang et al., 2010, 2023).

Sucrose serves as a main photosynthetic product in sugarcane and is the primary sugar transported from source to sink and storage tissues. The synthesis, transportation, and metabolism of sucrose involve a network of enzymes [such as Sucrose Phosphate Synthase (SPS), Sucrose Synthase (SuSy), invertases], sugar transporters (STs), transcription factor (TFs), protein kinases, and hormones (Khan et al., 2023). STs have a vital role in coordinating carbon efflux, facilitating the transport of sugars from photosynthetic organs (source) to storage organs (sink) (Lemoine, 2000). These STs are found in various plant species and consist of different gene families such as monosaccharide transporters (MSTs) and sucrose transporters (SUTs); the sugars will eventually be exported transporters (SWEET) and sucrose carriers (SUCs) (Slewinski et al., 2009; Lin et al., 2014). Additionally, TFs like AP2/ERF, NAC, GRF, and bZIP are known to play a significant role in sucrose synthesis and transport (Ma et al., 2019; Stein and Granot, 2019; Wang et al., 2022).

Research has identified ST gene families across various species, including dicots and monocots like Arabidopsis (*Arabidopsis thaliana*) (Büttner, 2007; Sauer, 2007), rice (*Oryza sativa*) (Aoki et al., 2003), tomato (*Solanum lycopersicum*) (Reuscher et al., 2014), pear (*Pyrus*) (Li et al., 2015), and grapes (*Vitis vinifera*) (Afoufa-Bastien et al., 2010). The *Saccharum spontaneum* genome has revealed the presence of the ST gene family, associated with the MFS, which plays a pivotal role in sugar transport (Zhang et al., 2018, 2021). The SUT and MST transporter families, powered by H+ ATPase pumps, regulate carbon allocation within plants, significantly impacting crop yield and nutritional value (Eom et al., 2015; Jeena et al., 2019).

This study marks the first identification of ST genes in *E. rufipilus* (diploid) and *S. officinarum* (polyploid) genomes, employing a bioinformatics approach to explore their physiochemical properties, chromosomal distribution, and evolutionary relationships. By analyzing the TFs that regulate the *SUT1-T1* gene in *S. officinarum* using Y1H assay, we identify potential TFs for improving sugarcane breeding. Additionally, investigating ST genes in *E. rufipilus* offers fundamental insights into gene evolution and the genetic framework of sugar transport and allocation, underscoring the complexity and potential within sugarcane genetics for agricultural advancement.

## Materials and methods

### Plant materials

*Saccharum* species, *E. rufipilus* and *S. officinarum*, studied as an experimental material. While, two species from the Andropogonae tribe, *S. spontaneum* and *S. bicolor*, are used as a reference species.

### Identification of ST protein in *E. rufipilus* and *S. officinarum*

ST genes in *E. rufipilus* and *S. officinarum* were initially identified by performing BLASTP (with a cutoff e-value 1e^−5^) search with already reported ST genes from *S. spontaneum* (Zhang et al., 2018). Distinguished ST genes were further annotated using the CDD batch search (Marchler-Bauer et al., 2015). Then, the candidate ST genes were confirmed through the PFAM (with e value <1e^−5^) database (Mistry et al., 2021), using HMMER software v3.2.1.

### Evolutionary relationship of ST gene family

ST gene sequences of *E. rufipilus* and *S. officinarum* were aligned with *S. spontaneum* and *S. bicolor* ST gene sequences using the maximum likelihood (ML) method. The ML tree was generated using MEGA, version 7.0, with a bootstrap value of 1,000 replicates and a “Poisson correction” model (Tamura et al., 2021). The results were then visualized in the interactive tree of life (iTOL) program for generating a phylogenetic tree (Letunic and Bork, 2019).

### Physical properties, conserved motifs, and gene structure analysis

To analyze the conserved motifs among all the ST genes of *E. rufipilus* and *S. officinarum*, protein sequences were submitted to the online MEME (motif-based sequence analysis tools) suite 4.11.1 program (http://meme.nbcr.net/mem/cgi-bin/mem.cgi) (Bailey et al., 2015). The following parameters were adjusted: maximum number of motifs 20, minimum and maximum length between 15 and 60, number of repetitions, any. The results were then visualized in the TBtools program (Chen et al., 2018). Gene structures were identified using DNA sequences and exons of ST genes displayed using the Gene Structure Display Server (GSDS) (Hu et al., 2015). Molecular weight (MW) and isoelectric point (pI) of each ST gene were determined using the ExPASy online tool (http://web.expasy.org/compute_pi/). Subcellular localization of ST proteins was predicted through WoLF PSORT (http://wolfpsort.hgc.jp) (Horton et al., 2007). The transmembrane domain from the amino acid sequences of ST proteins were predicted using TMHMM Server v.2.0 (http://www.cbs.dtu.dk/services/TMHMM/).

### Identification of *cis*-regulatory elements in the promoter region

The promoter region upstream of 2500 bp of transcription start site of each ST gene was extracted using TBtools. *cis*-regulatory elements (CRE) located in ST promoters were predicted using PlantCARE online database (http://bioinformatics.psb.ugent.be/webtools/plantcare/html/). The identified *cis*-regulatory elements were analyzed using the Simple Biosequence viewer function on TBtools.

### Duplication and chromosomal location of ST genes

ST gene positions on chromosomes were detected from the General Feature Format Files (GFF3), and karyotyping was executed in TBtools (Chen et al., 2018). The duplication pattern for ST genes and protein-coding genes was investigated by an all-vs-all local BLAST with an E-value <1e^−5^. BLAST results were imported into MCScanX (v0.8) software (http://chibba.pgml.uga.edu/mcscan2/). Whole Genome Duplication (WGD)/Segmental Duplication (SD), and Tandem Duplication (TD) were identified with default parameters (Wang et al., 2012). Orthologous ST genes between *S. spontaneum*, *E. rufipilus*, *S. officinarum* and *S. bicolor* were identified using the Dual Synteny Plot tool in TBtools. The coding sequences of ST gene pairs were subjected to synonymous (ks) and non-synonymous (ka) substitution ratio according to the Nei-Gojobori method (Kumar et al., 2016). If the ratio of Ka/Ks is greater than 1, it shows positive selection. When the value of Ka/Ks is equal to 1, it represents neutral selection. However, when the ratio of Ka/Ks is less than 1, it suggests negative or purifying selection. Additionally, we used the Ks values for estimation of duplication event time (T) in MYA (Million Years Ago). Here, T = Ks/(2 × 6.1 × 10^−9^) × 10^−6^ Mya (Gaut et al., 1996).

### Plant material and RNA extraction

We utilized different samples from 4–6 months old *E. rufipilus* and *S. officinarum* (B-48) tissues, including mature leaf, seedling stem, pre-mature stem, and mature stem. Total RNA from these harvested samples was extracted using a Quick-RNA™ Miniprep kit (Zymo Research, USA), according to the manufacturer’s recommendations.

### Transcriptome analysis by RNA-sequencing

We used RNA-seq data for *E. rufipilus* and *S. officinarum*, which included transcript abundance [Transcript Per Million (TPM)] from various developmental stages from our previous studies. Additionally, we incorporated RNA-seq data for *S. officinarum* that analyzed circadian rhythms, gradients of leaf development, and the effects of different hormones such as abscisic acid (ABA), gibberellin (GA), indole acetic acid (IAA), and ethylene (ETH) (Zhang et al., 2018, 2021; Hua et al., 2022; Wang et al., 2023).

Trimmed reads were aligned with the reference gene model of the *S. spontaneum* genome by Trinity with default settings (Grabherr et al., 2011). Transcript assembly was performed by Stringtie (Pertea et al., 2015), and TPM values were estimated by RSEM method (Li and Dewey, 2011).

### Plant growth and sugar treatment

*E. rufipilus* and *S. officinarum* stalks were grown in a greenhouse under growth conditions of 16h 30°C, 8h/22°C, and a relative humidity of 75%. Three sugar treatments (1% Sucrose, 1% Glucose, and 1% Fructose) were applied uniformly to the 6 months old seedlings. Each experiment was performed in triplicate. Leaf samples were collected after 8h of treatments and stored at −80°C for further processing.

### Confirmation of ST gene expression by quantitative RT-PCR

For quantitative RT-qPCR, about ≤1µg RNA was obtained from seedling stem, pre-mature stem, mature stem, and mature leaf samples. 5xPrimescript RT master mix (Takara Bio) was used for cDNA synthesis. RT-qPCR reaction comprising of the following reaction mixture in 20 µL solution: 10 µL of Master Mix (SYBR Green; Roche, Germany), 1 µL of template cDNA, 1 µL of forward and reverse primers each (10 µM), and water up to the final volume. Thermal cycling was as follows: initial denaturation at 95°C for 10 min, subjected by 40 cycles of denaturation at 95°C for 15 s, further annealing, and extension at 60°C for 60 s. For normalization of expression data, Actin and eEF-1a were used as reference genes. Each experiment was performed in three technical replicates. The relative expression level of each sample was calculated with the 2^-ΔΔCq^ method, as mentioned earlier (Akbar et al., 2021). A list of primers is mentioned in **Supplementary Table S1**.

### Transient protein expression and confocal microscopy

Full length CDS (*SUT1-T1*) without stop codon was amplified from *S. officinarum* cDNA (**Supplementary Table S1**). Amplified fragment of *SUT1-T1* was inserted into pCAMBIA1300-GFP vector. The vectors were then transformed into the *Agrobacterium tumefaciens* GV3101 strain. The bacterial suspensions with OD600 = 0.5 were inoculated into *Nicotiana benthamiana* leaves. FM™4-64 (N-(3-Triethylammoniumpropyl) was used as fluorescent dye. After 2–3 days of inoculation, leaf portions were excised from the injected area and observed under GFP fluorescence signals using a laser confocal microscope (LEICA TCS SP8).

### Yeast One-hybrid Assay

Yeast One-hybrid Assay (Y1H) screening was conducted using the Matchmaker Gold Yeast One-Hybrid Screening System (Clontech, 630489). The targeted sequences (bait) were cloned into the pAbAi vector. *SUT1-T1* promoter region (2000 bp upstream region from start codon) was amplified in four fragments [−1 to −500 (*cis*-1), −501 to −1000 (*cis*-2), −1001 to −1500 (*cis*-3), −1501 to −2000 (*cis*-4)] and cloned into pAbAi vector. The pAbAi-bait plasmids were then linearized with *Bst*B1 restriction enzyme and transformed into Y1H Gold yeast-competent cells. The colonies were screened on synthetic dextrose medium lacking uracil. The bait strains were then tested for Aureobasidin A (AbA) resistance, and the minimal inhibitory concentration of AbA was determined for bait strains. A cDNA library (prey) was generated by Ouyi Biomedical Technology Co., Ltd. (Shangai, China). The AD-prey vectors were co-transformed with bait-pAbAi plasmids and screened on SD/-Leu/AbA media. Potential binding partners were confirmed through sequencing. TFs were predicted through the Plant transcription factor database (PlantTFDB) (https://planttfdb.gao-lab.org/prediction.php). The primers used in Y1H assay are mentioned in **Supplementary Table S1**.

For the one-to-one interaction of *SUT1-T1* with LSD and NAC TFs, we extracted yeast plasmids from colonies confirmed by sequencing. The colonies were grown in YPDA medium, and plasmids were extracted using a yeast plasmid extraction kit (Solarbio, D1160). Due to the low copy number of yeast cells, we initially transformed 5 μL of the extracted yeast plasmids into *Escherichia coli* (*E.coli)* DH5α competent cells. Next, we transformed 100 ng of prey plasmid into yeast strain containing the corresponding bait plasmid to verify one-to-one interaction. The growth of the transformed yeast was analyzed on the SD/-Leu/AbA* selection medium.

## Results

### Identification of ST genes and their physical attributes

**Figure 1:**
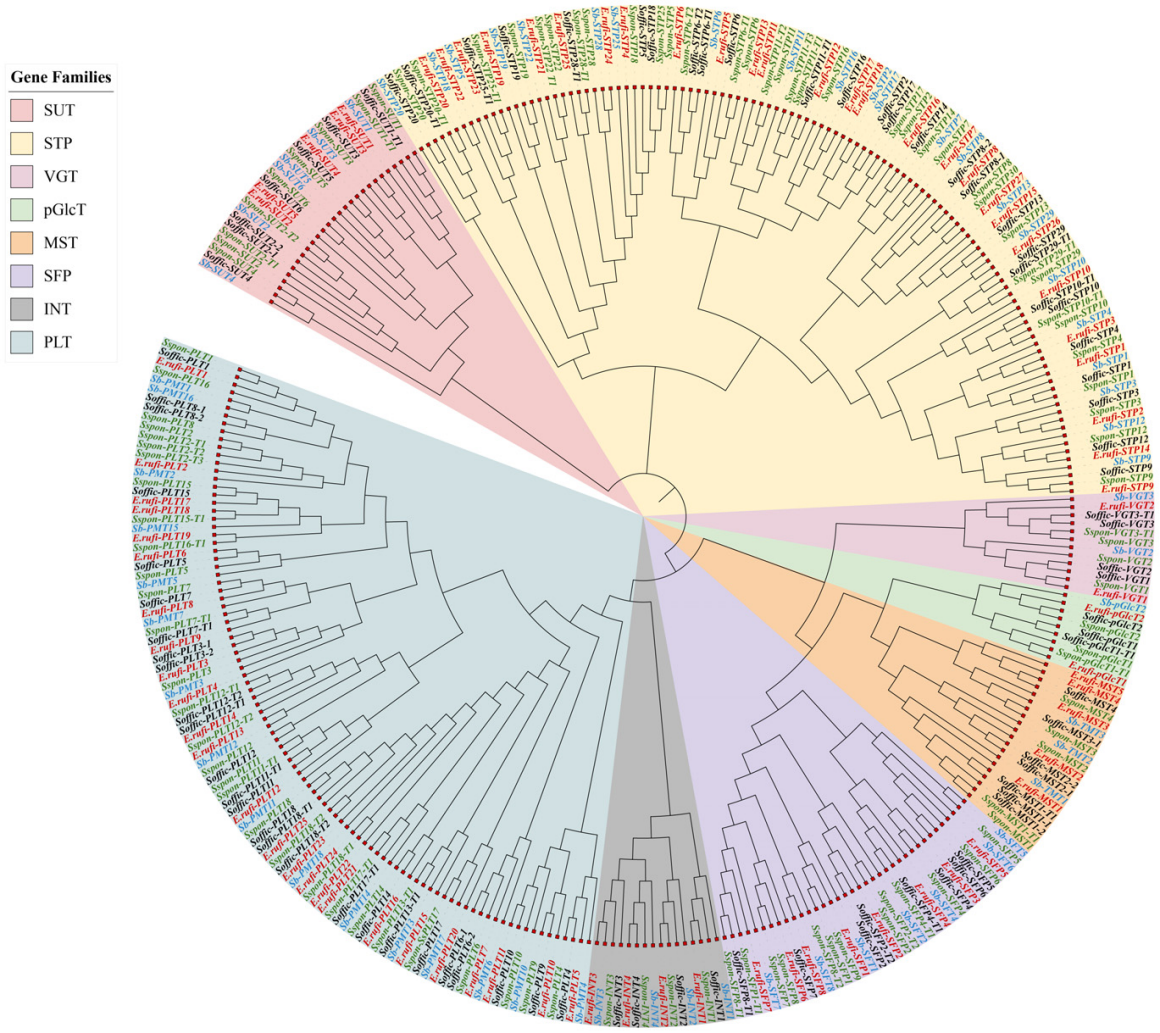
Phylogenetic tree of ST gene family of *S. officinarum*, *E. rufipilus*, *S. spontaneum* and *S. bicolor*.

A genome-wide search was performed using *E. rufipilus* and *S. officinarum* amino acid sequences as a query with BLASTp analysis, against the previously published ST genes in *S. spontaneum* (Zhang et al., 2021; Xiao et al., 2022). Query sequences were further confirmed through batch CDD search and PFAM. Subsequently, we identified 78 reliable ST genes from *E. rufipilus* and 86 ST genes from *S. officinarum*. These ST genes were further divided into eight subfamilies, including seven monosaccharide transporter families (VGT, INT, SFP, PLT, STP, MST, and pGlcT) and one sucrose transporter family (SUT). Consistent with earlier findings, phylogenetic analysis distributed the ST genes into eight distinct subfamilies (**Figure 1**) (Zhang et al., 2021; Xiao et al., 2022). All these ST genes were numbered according to their designated position in the evolutionary tree, with respective of *S. spontaneum* and *S. bicolor* ST genes. Using *S. spontaneum* ST genes as a reference, phylogenetic tree distinguished the 4 members in INT, 5 members each in MST and SUT, 2 members in pGlcT and VGT each, 25 members in PLT, 8 members in SFP, and *E. rufipilus* genome. In *S. officinarum* genome, 4 members were found in INT and VGT each, 7 members each in MST and SUT, 3 members in pGlcT, 38 members in PLT, and 8 members in SFP. Noticeably, the highest number of ST genes were found in the STP subfamily in *E. rufipilus*, which were 27 in numbers while in *S. officinarum* PLT subfamily contained the highest gene numbers, with 38 members.

ST gene families in *E. rufipilus* encode proteins with a length ranging from 348 amino acids (*Erufi. 02G037360-PLT23*) to 761 amino acids (*Erufi.10G030260-MST2*). Their molecular weight ranges from 37.14 KDa (*Erufi.02G037360-PLT23*) to 80.91 KDa (*Erufi.10G030260-MST2*). Isoelectric point (pI) predicted in the range of 4.74 pH (*Erufi.10G030260-MST2*) to 10 pH (*Erufi.09G014150-STP27*). Several trans-membrane domains are found to be between 6 and 12. Subcellular localization predicted by WoLF PSORT identified 40 ST genes located in plasmalemma, 27 in vacuole, 6 in chloroplast, and 5 in cytoplasm (**Supplementary Table S2A**). In *S. officinarum*, ST proteins range in length from 310 amino acids (*Soffic.09G0019430-6P-SFP7*) to 760 amino acids (*Soffic.06G0004520-3E-MST2*). Their molecular weight ranges from 33.109 KDa (*Soffic.09G0019430-6P-SFP7*) to 80.687 KDa (*Soffic.06G0004520-3E-MST2*). Iso-electric point (pI) is in the range of 4.76 pH (*Soffic.06G0004520-3E-MST2*) to 10.3 pH (*LAp.01H0033380-SFP4*). The number of transmembrane domain ranges between 6 and 12. Subcellular localization detected 45 ST genes in plasmalemma, 27 in vacuole, 7 in chloroplast, and 7 in cytoplasm (**Supplementary Table S2B**).

### Analysis of conserved motifs, coding sequences, and promoter sequences

**Figure 2:**
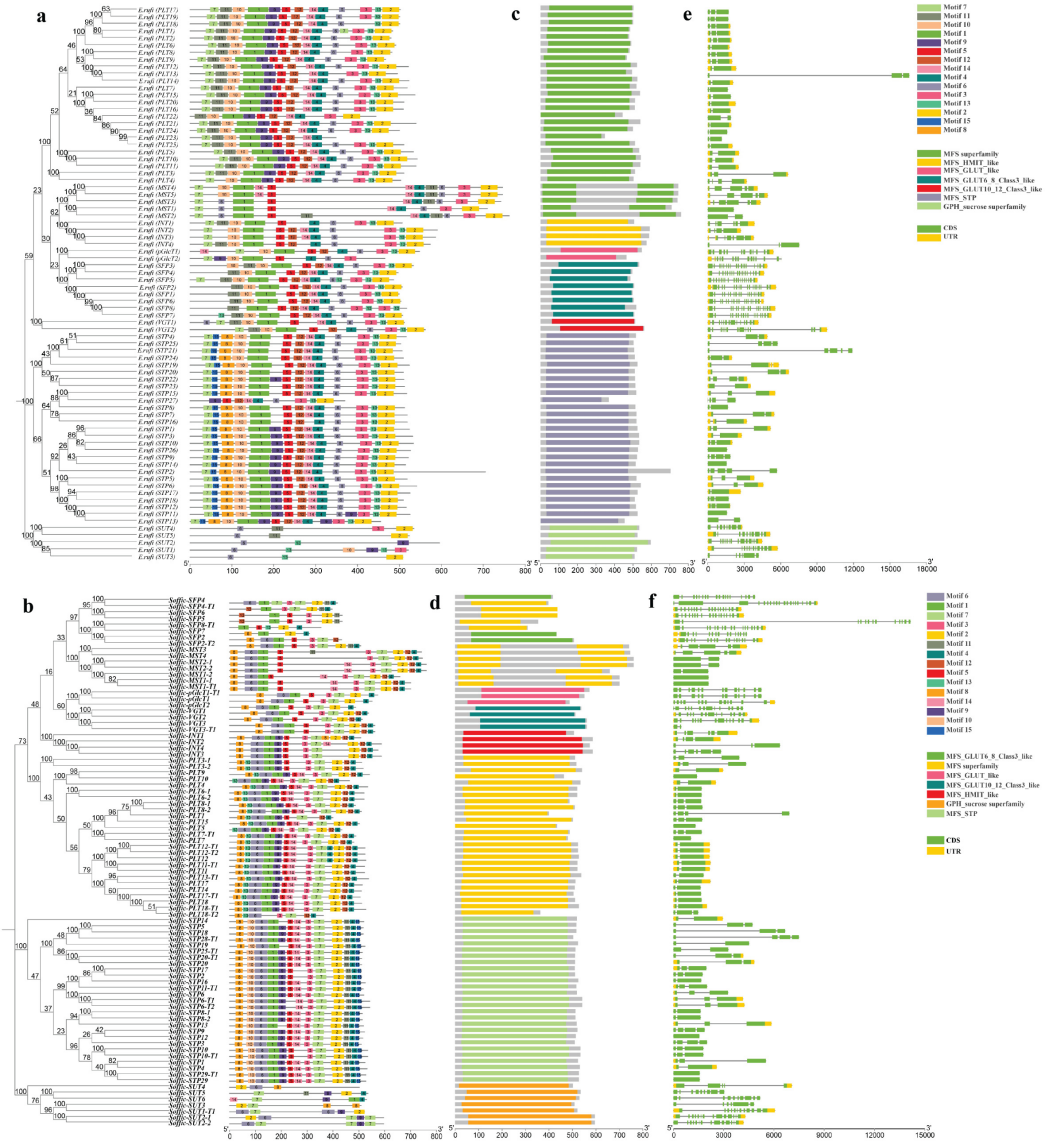
Phylogenetic tree, motif pattern, superfamilies, and gene structure of ST genes in *E rufipilus* and *S. officinarum* **(A, B)** The phylogenetic tree is contructed with Neighbor joining method with 1,000 replicates on each node, using full-length sequences of *E rufipilus* and *S. officinarum* ST proteins (number represents nucleotide substitutions per site). The amino acid motifs (numbered 1–15) are demonstrated in colored boxes, with black lones represented the protein length **(C, D)** Colored bars represent the distinct superfamilies in each sub family **(E, F)** Green and yellow rectangles represent the UTR (untranslated region) and exon, respectively.

Each member of the ST gene family has a unique sequence and distinct structural features. We identified conserved motifs to comprehend the structural diversity and evolutionary relationship. Importantly, few motifs were detected only in an individual family, which depicts that they are associated with specific functions. We detected 15 conserved motifs in ST genes of both species using the MEME suite (**Figures 2A, B**). In *E. rufipilus* and *S. officinarum*, commonly found motif at the N terminal is motif 7, except in SUT, while at C-terminal motif 2 is most common. Evidently, in the monosaccharide family number, the specificity of conserved motifs is highly comparable. However, some specific motifs are present or absent in each subfamily. Moreover, the number of conserved motifs in *STP* genes is 13 in both *E. rufipilus* and *S. officinarum.* Similarly, in the PLT subfamily conserved motifs are also found to be 13 in number in most of ST genes in both species. Furthermore, the number of conserved motifs in *SUT* genes ranges from 3 to 6 in *E. rufipilus* and 3 to 5 in *S. officinarum*. Conserved motifs depicted the origination of subfamilies from a common ancestor. However, slight differences showed the specificity and functional divergence of each subfamily.

Next, we detected seven superfamilies (MFS superfamily, MFS-GLUT10-12_Class3_like, MFS_GLUT_like, MFS_GLUT6_8_Class3-like, MFS_HMIT_like, MFS_STP, GPH_sucrose superfamily) in ST genes (**Figures 2C, D**). Each subfamily possesses a distinct superfamily. The PLT family was found to have MFS superfamily, VGT has MFS-GLUT10-12_Class3_like superfamily, MST also contained MFS superfamily, pGlcT is composed of MFS_GLUT_like superfamily, SFP has MFS_GLUT6_8_Class3-like superfamily, and INT encodes MFS_HMIT_like superfamily. Moreover, STP contained MFS_STP and SUT composed of the GPH_sucrose superfamily. The occurrence of these superfamilies contributed to the uniqueness and specific functionality of ST genes.

Regarding ST gene conservation, gene structures were illustrated with Gene Structure Display Server (GSDS). Each gene family was observed to display conserved exon and intron positions relative to domain position and structure (**Figures 2E, F**). To comprehend the evolutionary relationships and characteristics of ST sequences, the cDNA sequence of each ST gene was aligned with the genomic sequence. The majority of ST genes comprises only exons. In *E. rufipilus*, a total of 23 ST genes contain only exons, while 55 genes are composed of exons and untranslated regions (UTRs) as well. The number of exons per gene ranges from 2 to 18. Among 55 genes containing UTRs, only 16 genes have 3′ UTR and 39 have both 5′ and 3′ UTRs. In *S. officinarum*, 43 ST genes composed of only exons, with the number of exons per gene ranging from one to as many as 20 exons per gene.

For identification of *cis*-regulatory elements in the region, upstream 2.5 kb sequences from the start codon of each gene were submitted to PlantCARE. Promoter regions from ST genes revealed a number of *cis*-acting elements related to different functions (**Supplementary Figures S1A, B**). The majority of identified *cis*-acting elements were grouped into cellular function, stress response, light response, and hormonal regulation categories (**Table 1**). A number of *cis* elements are related to light responses such as ACE and AE box. Box4, GT1 motif, I-box, and Sp1 are present in both *S. officinarum* and *E. rufipilus*.

**Table 1:** Category wise list of cis-elements identified from 2.5kb upstream region of ST gene from *S. officinarum* and *E. rufipilus*.

Categories based on functions	Sequence	*Cis* element		Specific function	Reference
Cellular function	CATGCA	RY-repeat promoter motif	*S. officinarum*	Seed specific regulation	(Ezcurra et al., 2000)
			*E. rufipilus*		
Stress response	TTGACC	W-BOX	*S. officinarum*	Fungal elicitor responsive element, wound responsive	(Rushton et al., 2010)
			*E. rufipilus*		
	GT1-motif	Box-II promoter site	*S. officinarum*	Light responsive	(Le Gourrierec et al., 1999)
			*E. rufipilus*		
	I-box	I-box	*S. officinarum*	Light responsive	(Giuliano et al., 1988)
			*E. rufipilus*		
	G-box	G-box promoter motif	*S. officinarum*	Light responsive	(Menkens et al., 1995)
			*E. rufipilus*		
Light response	CCAAT box	–	*S. officinarum*	MYBHv1 binding site	(Liu et al., 2015)
			*E. rufipilus*		
	A-box	–	*S. officinarum*	Elicitor or light responsive	(Logemann et al., 1995)
			*E. rufipilus*		
	MBS	MYB3 binding promoter motif	*S. officinarum*	MYB binding site involved in drought inducibility	(Chen et al., 2002)
			*E. rufipilus*		
	HSE	HSE Binding site motif	*-*	Heat responsive	(Nover et al., 2001)
			*E. rufipilus*		
	BOX-S	SORLIP1	*-*	Light responsive	(Hudson and Quail, 2003)
			*E. rufipilus*		
Hormonal regulation	ABRE	ABRE like binding site motif	*S. officinarum*	ABA-regulated gene expression	(Hattori et al., 2002)
			*E. rufipilus*		
	TGACG	TGA1 binding site motif	*S. officinarum*	MeJA-responsive element, SA responsive element	(Kim et al., 1993)
			*E. rufipilus*		
	MBS	MYB3 binding promoter motif	*S. officinarum*	ABA-inducible	(Chen et al., 2002)
			*E. rufipilus*		

### Chromosomal distribution and synteny analysis of ST genes

**Figure 3:**
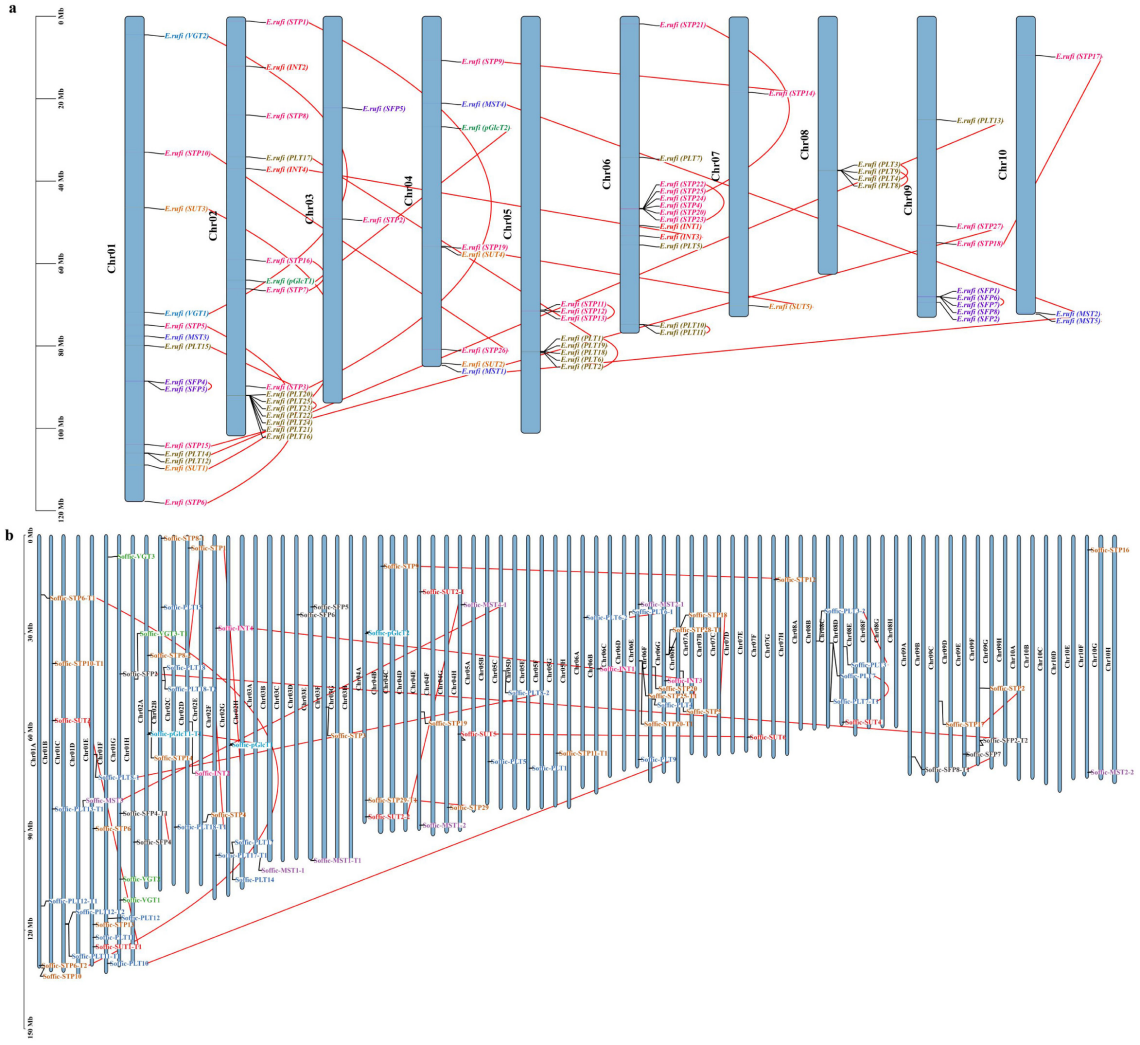
Chromosomal location of ST genes in **(A)** *E rufipilus* **(B)** *S. officinarum*. The colored rectangle bars represent the chromosomes of *E rufipilus* and *S. officinarum* and scale represent the chromosome length, the Chr 1–10 represent each corresponding chromosome, each gene sub family is represented by distinct color and red line represent the gene pairing.

We investigated the chromosomal distribution of ST genes on 10 chromosomes of *E. rufipilus* and *S. officinarum* (**Figures 3A, B**). We found that there is no substantial co-relation between the number of genes and chromosome length. The majority of the genes were found to be concentrated on Chr01 and Chr02 in *S. officinarum* while on Chr02 in *E. rufipilus*. All genes were indiscriminately distributed on chromosomes, with most genes located at the lower telomere.

Genomic sequence duplications, including WGD, TD, and SD, provide a genetic link for evolution (Xiao et al., 2022). The ST gene family duplication event was performed by MCScanX. Duplication analysis identified dispersed duplication (DD), WGD, or SD events in both genomes. WGD or SDs was found to be the primary reason for ST gene family expansion (**Supplementary Tables S3A, B**). In *E. rufipilus*, a total of 52 genes displayed WGD, or SD, and 26 genes were found to be DD. However, in *S. officinarum*, 58 genes depicted WGD or SD and 29 genes showed SD. This indicates that WGD or SD might be the major driving force in the evolution of the ST gene family. Additionally, 25 ST gene pairs were identified in *E. rufipilus* while 24 gene pairs were found in *S. officinarum*. These gene pairs were distributed on all 10 chromosomes (**Supplementary Figure S2**).

### Estimation of evolutionary rate and collinearity analysis

To further infer the evolutionary rate of ST genes, we calculated Ka/Ks (the ratio of synonymous and non-synonymous substitution rate). Ks values investigated positive (Darwinian) selection or negative (purifying) selection and duplication dates. ST gene pairs estimated to have undergone purifying selection. Duplication time was estimated to be in the range between 8.802 and 277.38 Mya in *E. rufipilus* and 0.182-90.444 Mya in *S. officinarum* (**Supplementary Tables S4A, B**). We performed collinearity analysis with *S. spontaneum* and *S. bicolor* to further identify homology between related species. All the ST genes belonging to *E. rufipilus* and *S. officinarum* showed collinearity with the syntenic region in *S. spontaneum* and *S. bicolor* (**Supplementary Figure S3**).

### Expression pattern of ST genes during different developmental stages

**Figure 4:**
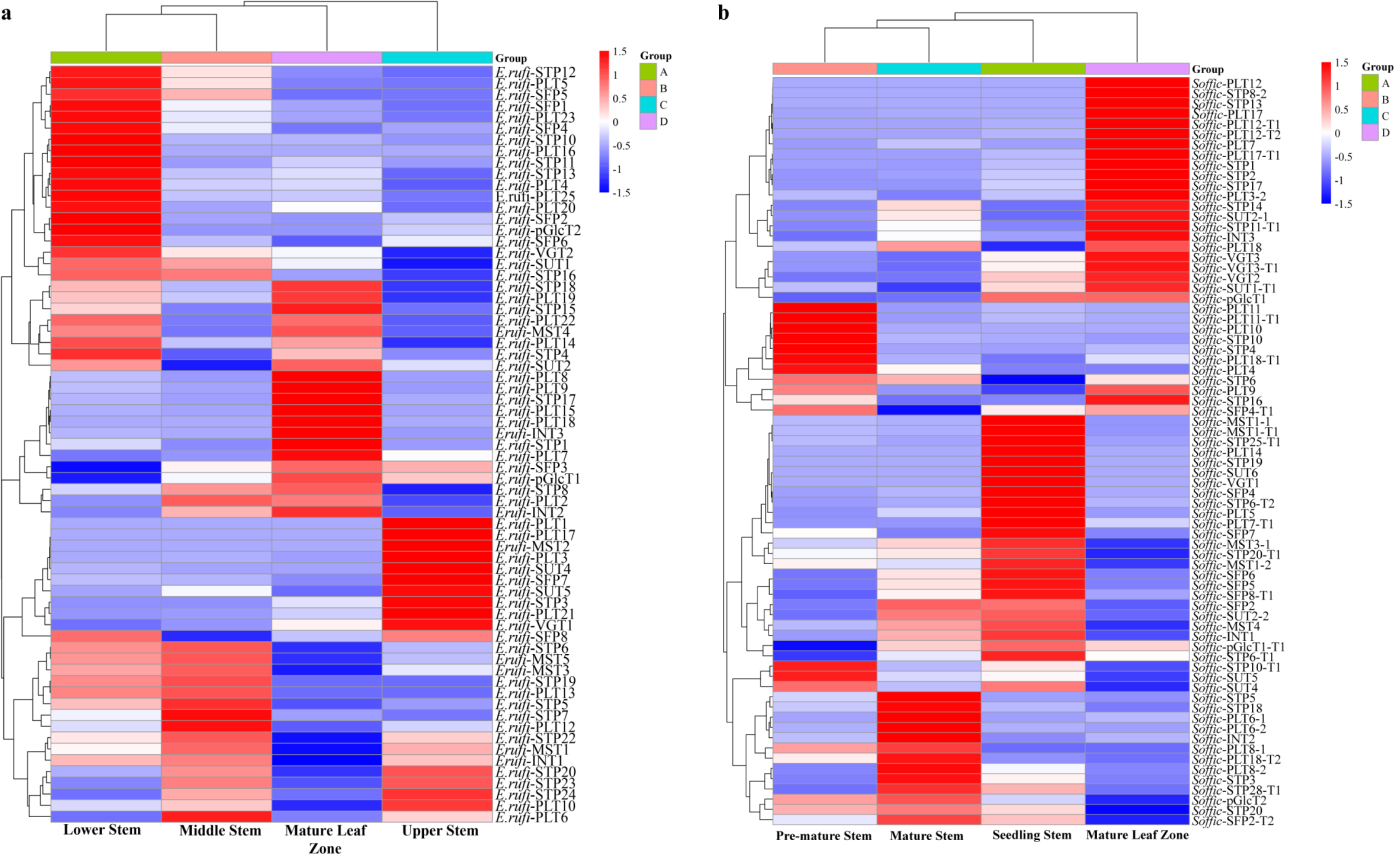
Gene expression analysis of ST genes **(A)** in different tissues (seedling stem, mature leaf, mature stem and pre-mature stem) of *E rufipilus* **(B)** in different tissues (seedling stem, pre-mature stem, mature stem, mature leaf) of *S. officinarum*.

To gain insights into the role of ST genes in sugar transport and mobilization, we analyzed the transcriptome profiles of ST gene expression in various tissues of *E. rufipilus* and *S. officinarum*. We found that the ST genes exhibited different expression patterns in different tissues. TPM values of selected ST genes were visualized using heatmaps. Specifically, we focused on the highly expressing genes in the mature leaf zone. In *E. rufipilus*, the following genes were up-regulated in the mature leaf zone: *SUT2*, *STP18*, *STP15*, *STP4*, *STP17*, *STP1*, *STP8*, *PLT2*, *PLT7*, *PLT18*, *PLT15*, *PLT9*, *PLT8*, *PLT14*, *PLT22*, *PLT19*, *SFP3*, *INT1*, *INT2*, and *MST4* (**Figure 4A**). However, in *S. officinarum*, the undermentioned genes were enhanced in the mature leaf zone: *SUT1-T1*, *SUT2*, *STP16*, *STP11-T1*, *STP17*, *STP2*, *STP1*, *STP13*, *STP8-2*, *PLT9*, *PLT18*, *PLT3-2*, *PLT17-T1*, *PLT7*, *PLT12-T2*, *PLT12-T1*, *PLT17*, *PLT12*, *SFP4-T1*, *INT3*, *VGT2*, *VGT3*, *VGT3-T1* (**Figure 4B**).

### Validation of gene expression pattern through RT-qPCR

To verify the accuracy of the RNA seq data, we conducted RT-qPCR on different tissue samples from *E. rufipilus* and *S. officinarum*. The RT-qPCR results showed that *SUT1* is highly expressed in the mature stem of *E. rufipilus* and mature leaf of *S. officinarum*. However, *INT1* exhibits high expression in the pre-mature stem of *E. rufipilus* and mature leaf of *S. officinarum*. Additionally, in *S. officinarum*, there is higher expression of *STP4* and *PLT11* in pre-mature stem. In *E. rufipilus*, *PLT15* and *STP23* depicted enhanced expression in mature leaf. Conclusively, we observed consistent trends in the relative expression of selected ST genes and their corresponding FPKM values (**Supplementary Figure S4**).

### Expression pattern of ST genes during different sugar stresses and subcellular localization of *SUT1-T1*

**Figure 5:**
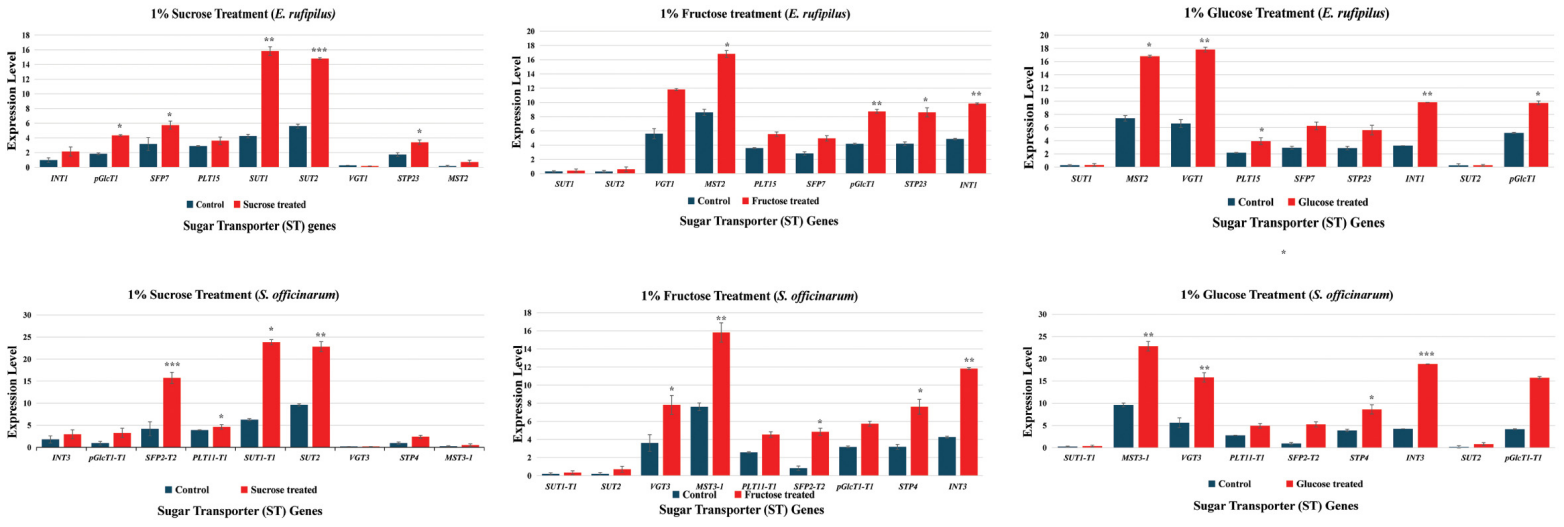
Relative gene expression level of nine selected ST genes (*INT1*, *pGlcT1*, *SFP7*, *PLT15*, *SUT1*, *SUT2*, *VGT1*, *STP23*, *MST5*) in *E. rufipilus* and *S. officinarum*, under different sugar stress (1% sucrose, 1% glucose, 1% fructose). * (p-value <0.05), ** (p-value <0.01), *** (p-value <0.001).

To better understand the role of ST genes, we treated the *S. officinarum* and *E. rufipilus* leaves with different sugar solutions and analyze their expression pattern through RT-qPCR. Expression data was obtained from 4 to 6 months old leaves of *S. officinarum* and *E. rufipilus*, treated with 1% solution of sucrose, glucose, and fructose. The expression level of ST genes showed variation during different sugar treatment (**Figure 5**).

During 1% sucrose treatment, *SUT1* and *SUT2* showed enhanced expression in both *E. rufipilus* and *S. officinarum* leaves. Additionally, *SFP* expression was also found to be relatively higher in sucrose-treated *S. officinarum* leaves. Furthermore, the 1% glucose treatment regulates expression of *MST*, *VGT*, *INT*, and *pGlcT* gene levels in both studied species as compared to their wild-type counterparts. Interestingly, the 1% fructose treatment resulted in increased expression of *MST*, *VGT*, and *INT* in both *E. rufipilus* and *S. officinarum*. We also observed moderately higher expression levels of *PLT*, *SFP*, *pGlcT*, and *STP* in both species compared to their wild-type counterparts. Notably, the expression levels of *SUT1* and *SUT2* were remarkably low during the glucose and fructose treatments.

**Figure 6:**
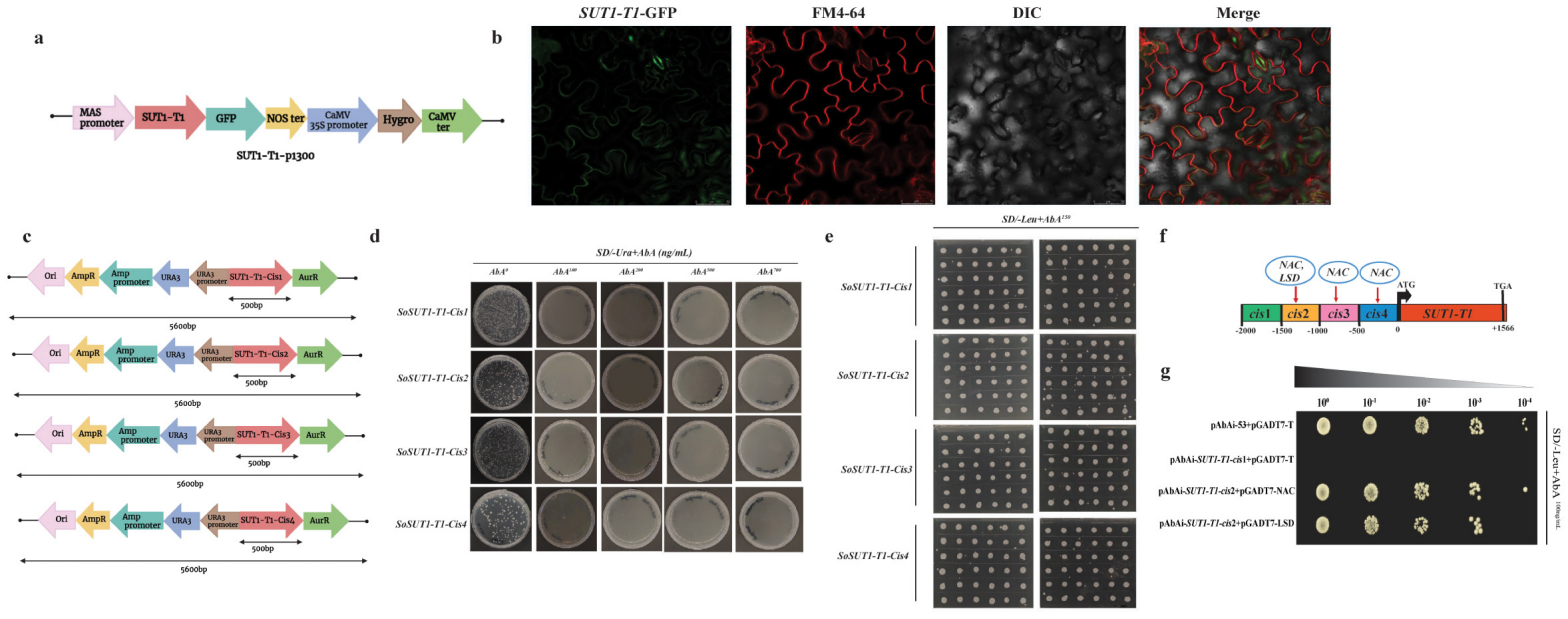
Sub cellular localization of *SUT1-T1* gene in *Nicotiana benthamiana* leaves **(A)** Diagrammatic representation of *SUT1-T1* gene in p1300 binary vector **(B)** Confocal microscopy of *SUT1-T1* gene in *N. benthamiana* leaf epidermis. Scale bar used 50μm **(C)** Schematic representation of four *cis*-*SUT1-T1* fragments in AbAi vector that were used as baits in yeast one hybrid (Y1H) screening **(D)** Growth of yeast on SD/-Ura+AbA medium for primary screening. AbA^0^ without AbA antibiotic, AbA^100^: 100ng/mL, AbA^200^: 200 ng/mL, AbA^500^: 500 ng/mL, AbA^700^: 700 ng/mL **(E)** Growth of yeast on SD/-Leu+AbA^150^ medium for secondary screening **(F)** Schematic representation of the identified TFs in the promoter region of the *SUT1-T1* gene **(G)** Yeast one to one verification. PAbA53_pGADT7-T is a positive control and pAbAi-*SUT1-T1-cis*1*+*pGADT7-T is a negative control. Top bar indicates the serial dilutions.

Based on the analysis of expression data, we identified *SUT1-T1* with high expression in the mature leaf zone of *S. officinarum*. We selected this gene to investigate its subcellular location. Confocal microscopy determines that *SUT1-T1* is located in the plasma membrane (**Figures 6A, B**). This finding is consistent with the prediction of the WoLF PSORT tool, which also identified the plasma membrane as the location of the *SUT1-T1* gene.

### Identification of TFs in *SUT1-T1* promoter region of *S. officinarum*

In order to identify the potential TFs that bind to the *SUT1-T1* promoter region, we divided the 2000 bp promoter region into smaller fragments of 500 bp (**Figure 6C**). These fragments were referred to as bait and were used to screen sugarcane cDNA libraries using the Y1H system. The activity of the bait fragments was repressed using 100 ng ml^−1^ of AbA (**Figure 6D**). We determined that the minimal inhibitory concentration of AbA for screening the sugarcane cDNA library was 100 ng ml^−1^. A total of 100 clones for each fragment were grown on SD/-Leu/+AbA (150 ng ml^−1^) medium and screened for TFs prediction (**Figure 6E**). Of the total clones screened, only four showed potential TFs. According to Plant TFDB, NAC (*Soffic*.10G0023830-1A) and LSD (*Soffic*.07G0003300-4D) were identified as potential TFs (**Figure 6F**). Next, the one-to-one interaction between NAC and LSD TFs with the *SUT-T1*-*cis*2 promoter region confirms that both TFs interact with the gene promoter region (**Figure 6G**).

**Figure 7:**
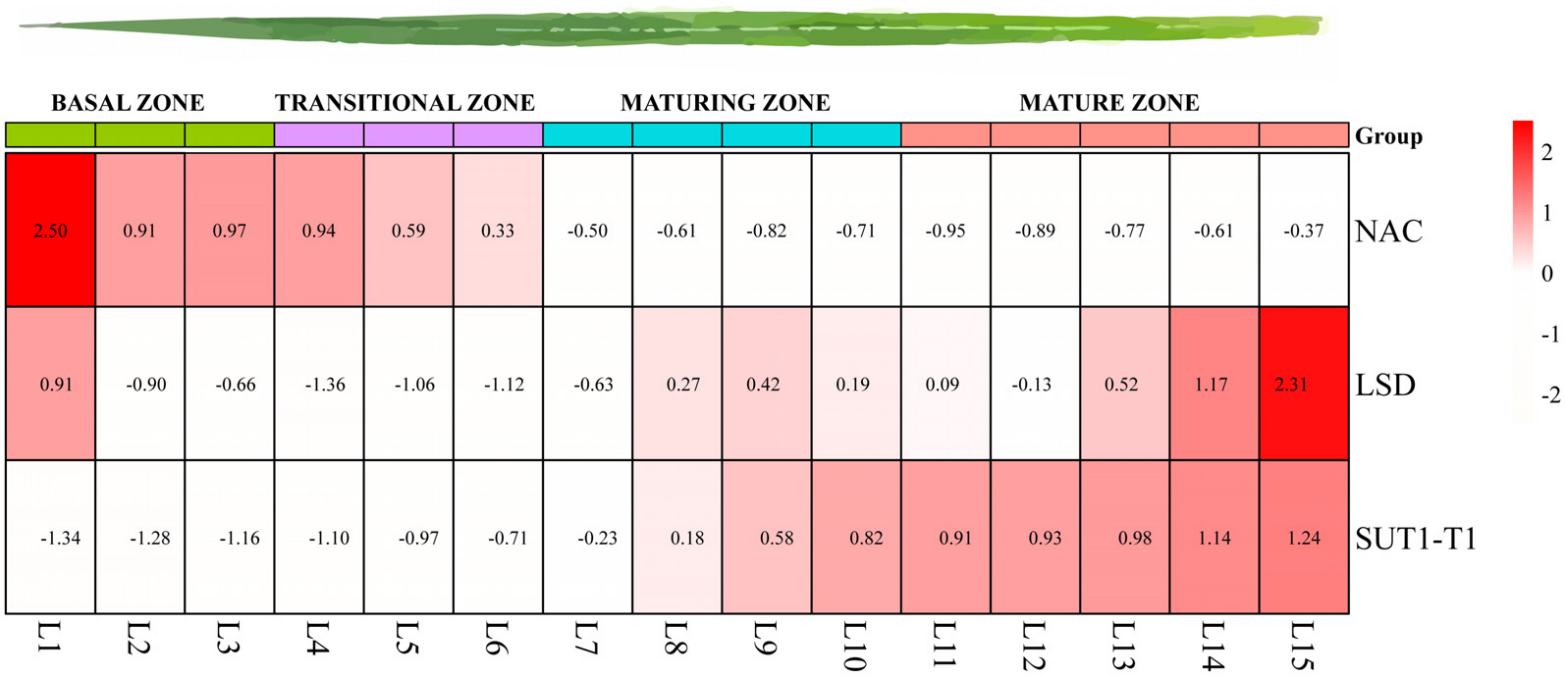
Expression patterns of *SUT1-T1* and its potential upstream TFs across gradient developmental leaves based on TPM.

To investigate the expression patterns of TFs identified in the Y1H experiment, RNA-seq analyses were performed in *S. officinarum*, focusing on various developmental stages, diurnal cycles, and leaf development. We compared the expression patterns of NAC and LSD TFs with *SUT1-T1* by analyzing the seedling leaves. Seedling leaves, measuring 15 cm in length, were divided into 15 equal segments. The expression of *SUT1-T1* consistently increased from the basal to the mature zone. LSD exhibited a positive correlation with *SUT1-T1*, while NAC depicted a negative expression pattern (**Figure 7**).

To investigate how TFs regulate *SUT1-T1* at various developmental stages, we measured their transcript abundance in mature tissues, specifically in leaves and stems. Both TFs, NAC and LSD, exhibited higher expression levels in mature stem-internode 9. In contrast, *SUT1-T1* was expressed in seedling leaves. This indicates that NAC and LSD TFs negatively regulate the *SUT1-T1* gene (**Supplementary Figure S5A**). Next, we compared the expression pattern of TFs on *SUT1-T1* during day-night rhythm in *S. officinarum* (**Supplementary Figure S5B**). *SUT1-T1* showed higher expression levels during the daytime (from 6:00 to 10:00) and decreased expression for the reminder of day. LSD displayed enhanced expression in the morning (at 10:00) while NAC showed increased expression from 14:00 until midnight.

The expression patterns of NAC in the leaves and stems of *S. officinarum* were assessed following treatments with different hormones (IAA, ETH, GA, ABA) at various time points (**Supplementary Figure S5C**). NAC was found to be highly expressed in the leaves after ABA treatment and in the stem after 24h. However, NAC expression was higher in the stem at 48h and 96h after ETH treatment. GA increased NAC expression at all time points in the stem, while in the leaves, it was only elevated after 96h. IAA induced a strong NAC expression in the leaves after 48h of treatment. However, ETH and ABA had no effect on LSD expression, whereas GA and IAA enhanced LSD expression in all treated samples. *SUT1-T1* expression is high in stems treated with ABA for 48 and 96 h. ETH increases *SUT1-T1* expression in the 48h leaf, 24h stem, and 96h stem samples. GA enhances *SUT1-T1* expression in the 24h and 96h leaf samples, as well as the 24h stem. Conversely, IAA enhanced the expression of *SUT1-T1* in all treated leaf samples. Overall, there is no correlation observed in the expression patterns of NAC, LSD, and *SUT1-T1* under various hormone treatments. Additionally, we have found that the expression pattern of the selected genes depicted a consistent trend between transcriptome data and RT-qPCR values (**Supplementary Figure S6**).

## Discussion

Whole genome sequencing (WGS) offers opportunities to identify and functionally analyze the gene families in plants. However, it is troublesome to study polyploid genomes, as they are composed of homeologous sub-genomes, and allocating each sequence to respective chromosomes is challenging. Previously in the genus *Saccharum*, genomes of *S. spontaneum* and *E. rufipilus* have been published, providing detailed information about their genome architecture and origins (Zhang et al., 2018; Wang et al., 2023). Additionally, *E. rufipilus* (diploid) genome could be used as a reference for genomics analysis in sugarcane (Wang et al., 2023).

Gene duplication events are important for evolution and the expansion of gene families (Moore and Purugganan, 2003). Previous studies have shown that multigene families have evolved in the *Saccharum* genome by two WGD events (Zhang et al., 2019). WGD or SD suggests that a gene might arise from WGD or SD. This is one of the highest duplication events in angiosperms, which have underwent through minimum one WGD event (Jiao et al., 2011). During DD events, genes might arise from transposition such as replicative transposition, non-replicative transposition, or conservative transposition (Taylor and Raes, 2005). In our study, WGD/SD occurred in 52 and 58 ST genes of *E. rufipilus* and *S. officinarum*, respectively. Meanwhile, 26 and 29 ST genes showed DD events in *E. rufipilus* and *S. officinarum*, respectively. This suggests that WGD/SD is the major driving force behind the evolution of ST gene families in both studied species.

Sugars are transported from leaves to stems or sink tissues with various ST proteins or genes. Various STs are located inside the cell to distribute sugar to different compartments. We compared the expression pattern of various ST genes across different tissues in both species (**Figure 4**). Plastid sugar transporters (*pGlcT*) are located on the inner-envelop membrane of plastid, which function in starch mobilization. Previously, *pGlcT2* expression was observed in the seedlings and early stages of plant growth (Valifard et al., 2023). However, analysis of RNA-seq data revealed that *pGlcT2* exhibits a high expression level in the mature stem of *S. officinarum* and *E. rufipilus*. *PLT* members are located on the plasma membrane and are responsible for the transportation of hexoses and various sugar alcohols (Klepek et al., 2005). Previously, *PLT11* and *PLT11_T1* showed high expression levels in sclerenchyma and parenchyma cells of mature stalk and resulted in high sugar content in *S. officinarum* (Zhang et al., 2021). Based on the RNA-expression data, it has been determined that *PLT11* and *PLT11-T1* are found to be higher in the pre-mature stem of *S. officinarum.*

Glucose and fructose molecules are transported inside vacuoles with *VGT* genes. In both studied species, *VGT1* has been found to have high expression levels in the seedling stems. Earlier research has shown that *VGT3* exhibits high expression level in the leaves of *S. officinarum* and *S. lycopersicum* (Reuscher et al., 2014). *SUT*s, belonging to the MFS, are responsible for the long-distance transport of sucrose in plants. In *Saccharum*, SUT proteins primarily bind with the disaccharide sucrose (Wendler et al., 1991). In *S. officinarum*, the expression of *SUT2* and *SUT1-T1* is high in mature leaf, while in *E. rufipilus*, *SUT2* is specifically enhanced in mature leaf. Consistently, transcription activity of *SUT1* and *SUT2* was also found to be higher in mature leaves during different sugar treatment (**Figure 5**).

TFs play a crucial role in regulating various physiological activities in plants by binding to conserved sites within promoter regions of target genes. In the upstream 2 kb promoter sequence of *SUT1-T1*, *cis*-element prediction has revealed the presence of binding sites for NAC and LSD TFs. NAC TFs play a role in various biological processes that control plant growth and development, such as responding to external stress (Nuruzzaman et al., 2013), forming flower organs (Sablowski and Meyerowitz, 1998), forming shoot and root apical meristems (Souer et al., 1996), developing lateral roots, and regulating senescence (Xie et al., 2000; Podzimska-Sroka et al., 2015). The role of LSD TFs in *Arabidopsis* is to negatively regulate a plant cell death pathway. LSD1 triggers the systemic acquired resistance (SAR) response to both biotic and abiotic stresses (Dietrich et al., 1997). In *S. spontaneum*, 115 NAC genes have been identified that are involved in responding to biotic and abiotic stresses (Shen et al., 2022). However, the role of LSD in *Saccharum* has not yet been studied.

Based on the TFs identified in the Y1H assay, a series of transcriptome analyses were conducted to investigate their relationship with *SUT1-T1*. In this study, *SUT1-T1* is found to be highly expressed in seedling leaves (**Supplementary Figure S5**). The expression patterns of NAC and LSD, in conjuction with the leaf gradient, exhibited an inverse relationship with that of *SUT1-T1*. Expression level of *SUT1-T1* is peaked at 08:00 and begins to decline by 10:00; Wile LSD expression reaches its peak at 10:00. Additionally, the diurnal expression pattern of NAC is negatively correlated with that of *SUT1-T1*. Therefore, we hypothesize that NAC and LSD in *S. officinarum* respond to sucrose transport by exhibiting a negative regulatory relationship with *SUT1-T1*.

Overall, regarding the function of *SUT1-T1* and its potential TFs, we proposed a regulatory network for *SUT1-T1* based on gene expression profiles (**Supplementary Figure S7**). *SUT1-T1* is primarily active in mature leaves and seedling leaves, playing a role in sucrose transportation from source to sink tissues. In the source tissue, *SUT1-T1* is highly active but negatively regulated by NAC and LSD. In contrast, *SUT1-T1* is less active in sink tissues, where NAC and LSD are highly expressed. Additionally, *SUT1-T1* shows increased activity during daytime, as part of the circadian rhythm. However, this speculation still requires verification through additional approaches.

## Conclusion

In this study, we performed a comprehensive analysis of the ST gene family in *E. rufipilus* and *S. officinarum.* This study highlights the key genes, their location, interaction, and expression pattern. Additionally, our transcriptome analysis of different tissues and sugar stress provided insights into the key genes involved in the sugar transportation pathway. We also discovered that NAC and LSD TFs have the ability to bind with the *SUT1-T1* promoter. Transcriptome analyses of TFs across developmental gradient leaves, various time points during circadian cycles, and stems, and leaves at different growth stages reveal potential expression patterns and regulatory networks between these TFs and *SUT1-T1*. The data we generated will be valuable in understanding the ST gene families in the *Saccharum* genus. These fundamental results will be beneficial in identifying the key ST genes in other monocots, which can be utilized in plant engineering strategies.

## Data Availability

Publicly available datasets were analyzed in this study. This data can be found here:The RNA-seq data for *E. rufipilus* is available in the National Genomics Data Center (NGDC) under Bioproject accession PRJCA014818. The RNA-seq data for *S. officinarum* can be accessed through the National Center for Biotechnology Information (NCBI) under Bioproject ID PRJNA1200917.

## Supplementary material

Supplementary Figure S1
*Cis*-acting elements of the promoter region (upstream 2500 bp) of ST genes in **(A)** *E. rufipilus* and **(B)** *S. officinarum*.

Supplementary Figure S2 Collinearity of ST genes in **(A)** *E. rufipilus* and **(B)** *S. officinarum*. The red lines indicate the duplicated ST gene pairs. The chromosome number is shown inside each chromosome.

Supplementary Figure S3 Synteny analysis of ST genes between *E. rufipilus*, *S. officinarum*, *S. bicolor* and *S. spontaneum*. The gray region represents synteny blocks in the wide genome of four species, while colored lines represent the orthologous relationship of ST genes.

Supplementary Figure S4 Co-relation analysis of RT-qPCR data and gene expression data **(A)** in *E. rufipilus* **(B)** *S. officinarum.* Green bars represent the relative expression level of each gene in various tissues and red line indicates the positive co-relation of RNA-seq data with RT-qPCR data.

Supplementary Figure S5 Expression pattern of *SUT1-T1* and its potential upstream TFs were analyzed across multiple tissues, circadian rhythms and hormonal treatments, based on TPM.

Supplementary Figure S6 RT-qPCR verification of LSD **(A)** NAC **(B)** *SUT1-T1* **(C)** expression pattern in gradient developing leaves segments.

Supplementary Figure S7 Schematic representation of the expression pattern of LSD, NAC and *SUT1-T1* at different stages of *S. officinarum*, during both daytime and night time. Red indicates higher expression levels, white and yellow indicates lower expression levels.
