# Supplementary figures and images for: Genome-wide analysis of sugar transporter gene family in Erianthus rufipilus and Saccharum officinarum, expression profiling and identification of transcription factors
Source: Front Plant Sci. 2025 Jan 9;15:1502649. doi: 10.3389/fpls.2024.1502649 (PMC11755103; doi:10.3389/fpls.2024.1502649)

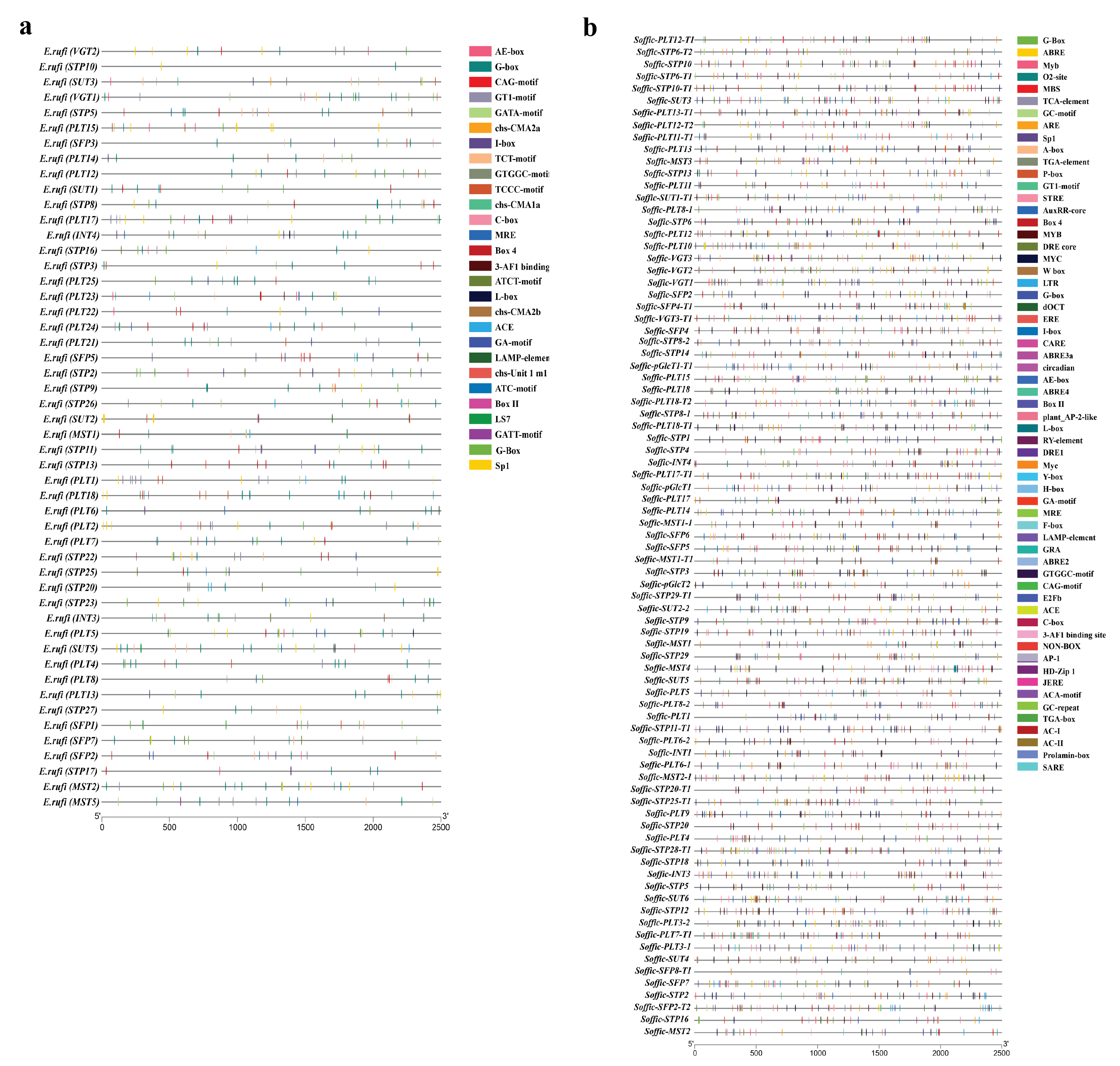

Supplement: Supplementary Figure S1 — Cis-acting elements of the promoter region (upstream 2500 bp) of ST genes in (A) E. rufipilus and (B) S. officinarum. [file Image1.png]

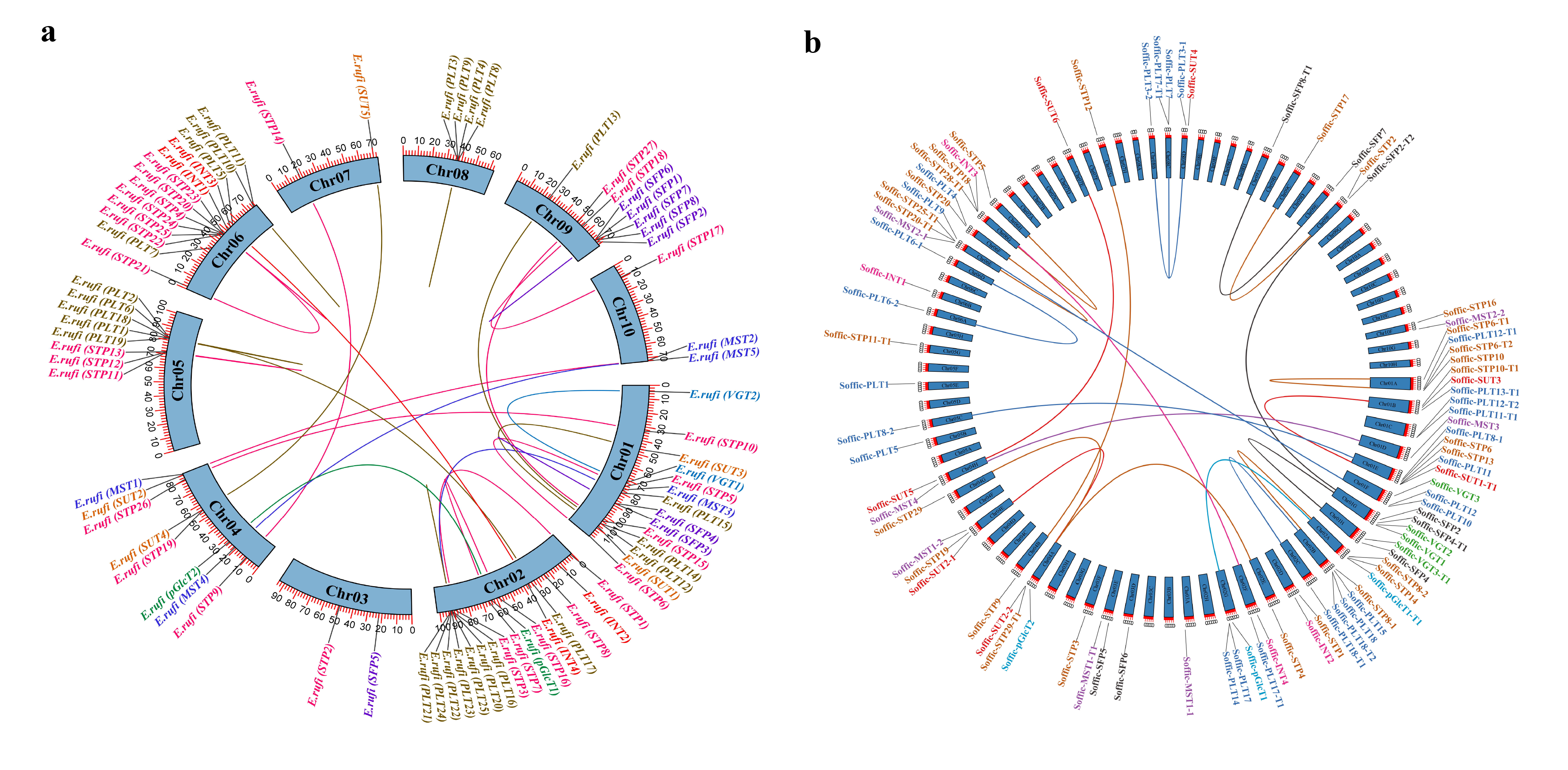

Supplement: Supplementary Figure S2 — Collinearity of ST genes in (A) E. rufipilus and (B) S. officinarum. The red lines indicate the duplicated ST gene pairs. The chromosome number is shown inside each chromosome. [file Image2.png]

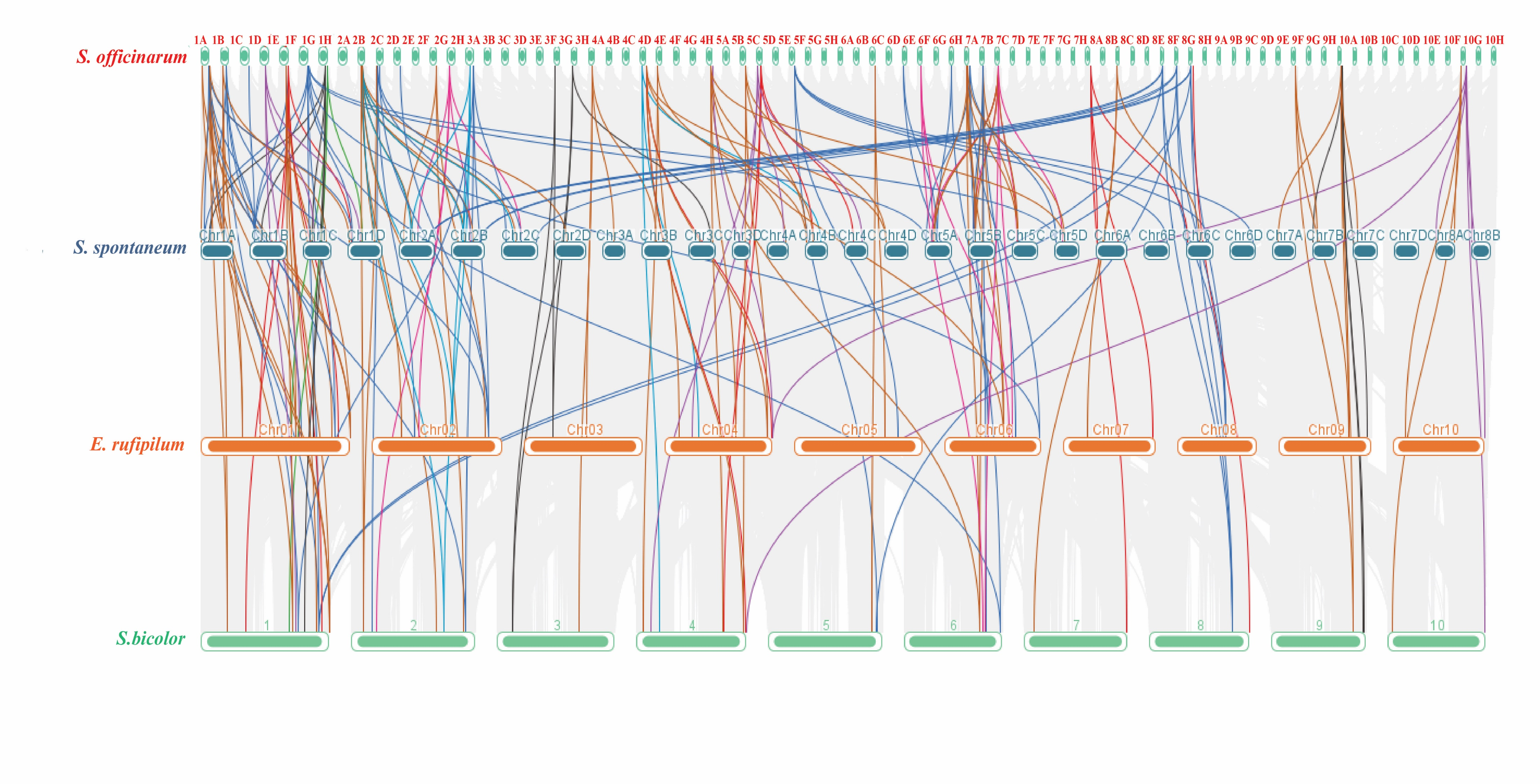

Supplement: Supplementary Figure S3 — Synteny analysis of ST genes between E. rufipilus, S. officinarum, S. bicolor and S. spontaneum. The gray region represents synteny blocks in the wide genome of four species, while colored lines represent the orthologous relationship of ST genes. [file Image3.png]

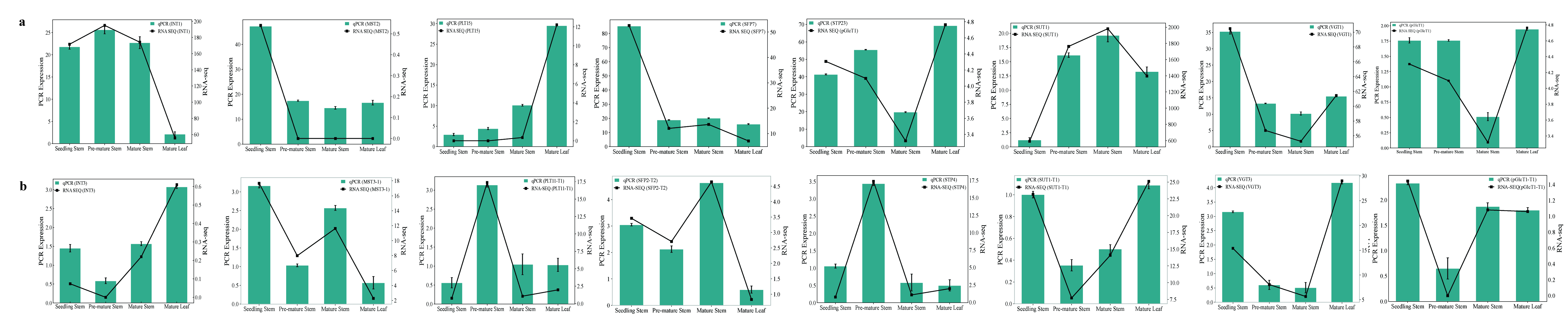

Supplement: Supplementary Figure S4 — Co-relation analysis of RT-qPCR data and gene expression data (A) in E. rufipilus (B) S. officinarum. Green bars represent the relative expression level of each gene in various tissues and red line indicates the positive co-relation of RNA-seq data with RT-qPCR data. [file Image4.tif]

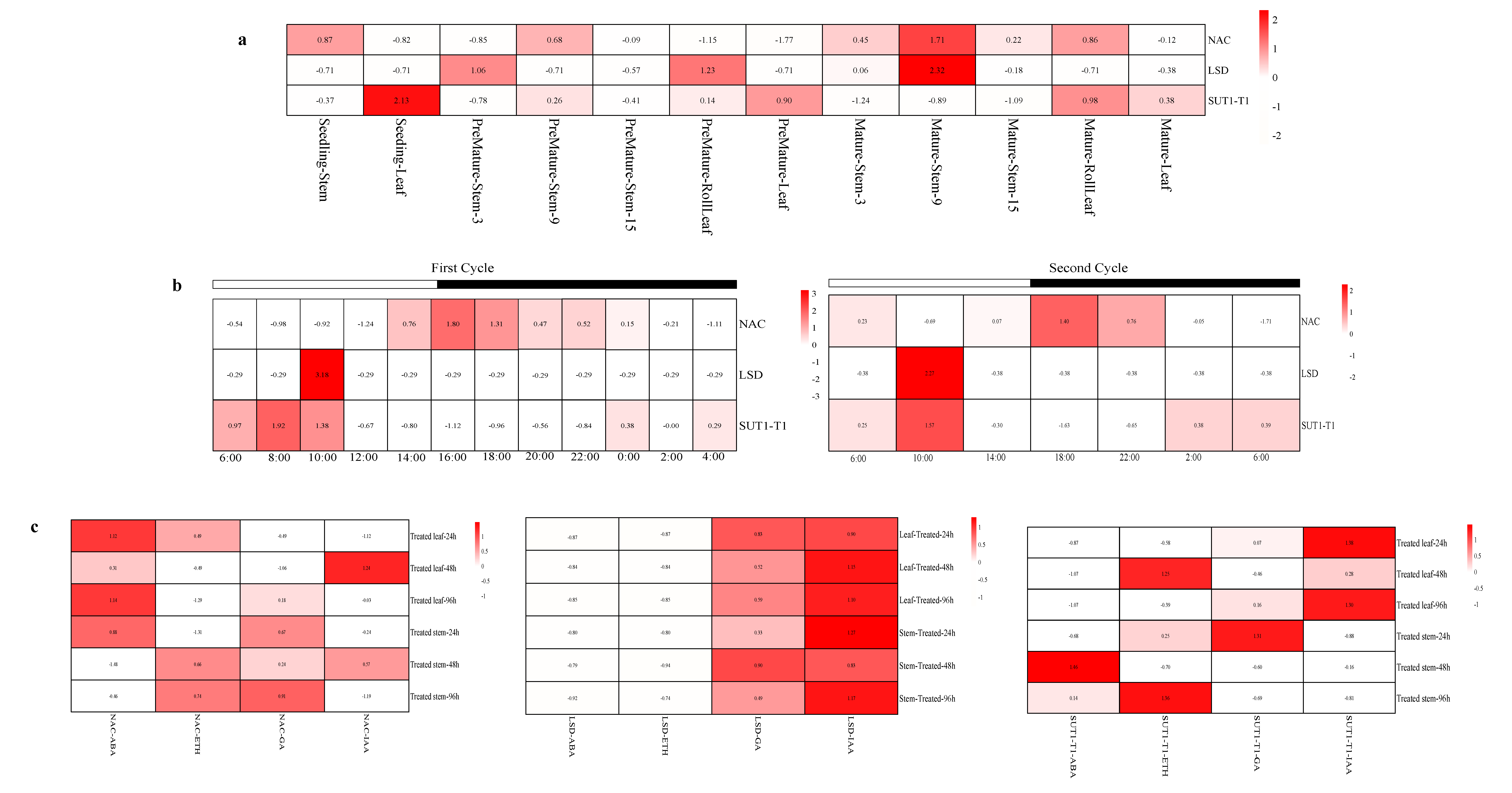

Supplement: Supplementary Figure S5 — Expression pattern of SUT1-T1 and its potential upstream TFs were analyzed across multiple tissues, circadian rhythms and hormonal treatments, based on TPM. [file Image5.png]

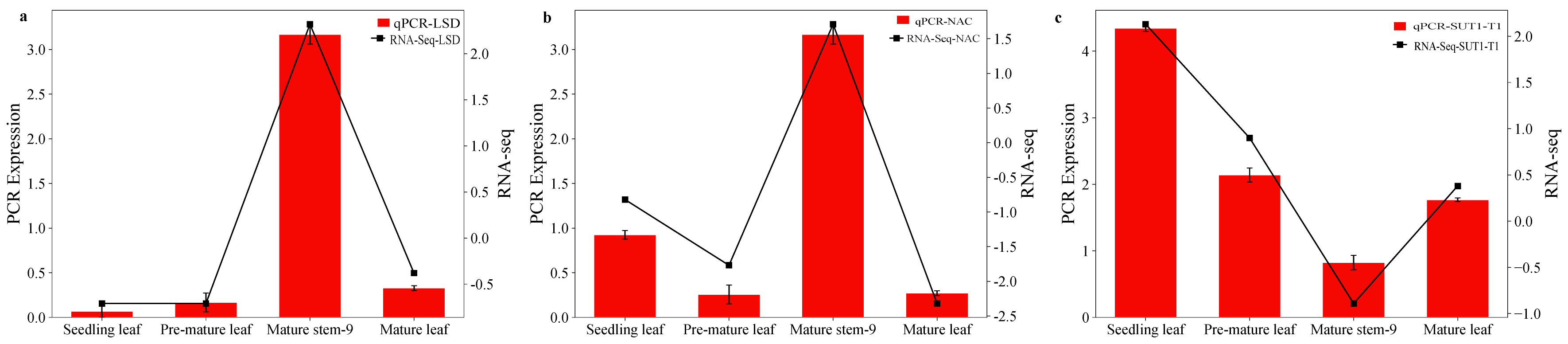

Supplement: Supplementary Figure S6 — RT-qPCR verification of LSD (A) NAC (B) SUT1-T1 (C) expression pattern in gradient developing leaves segments. [file Image6.tif]

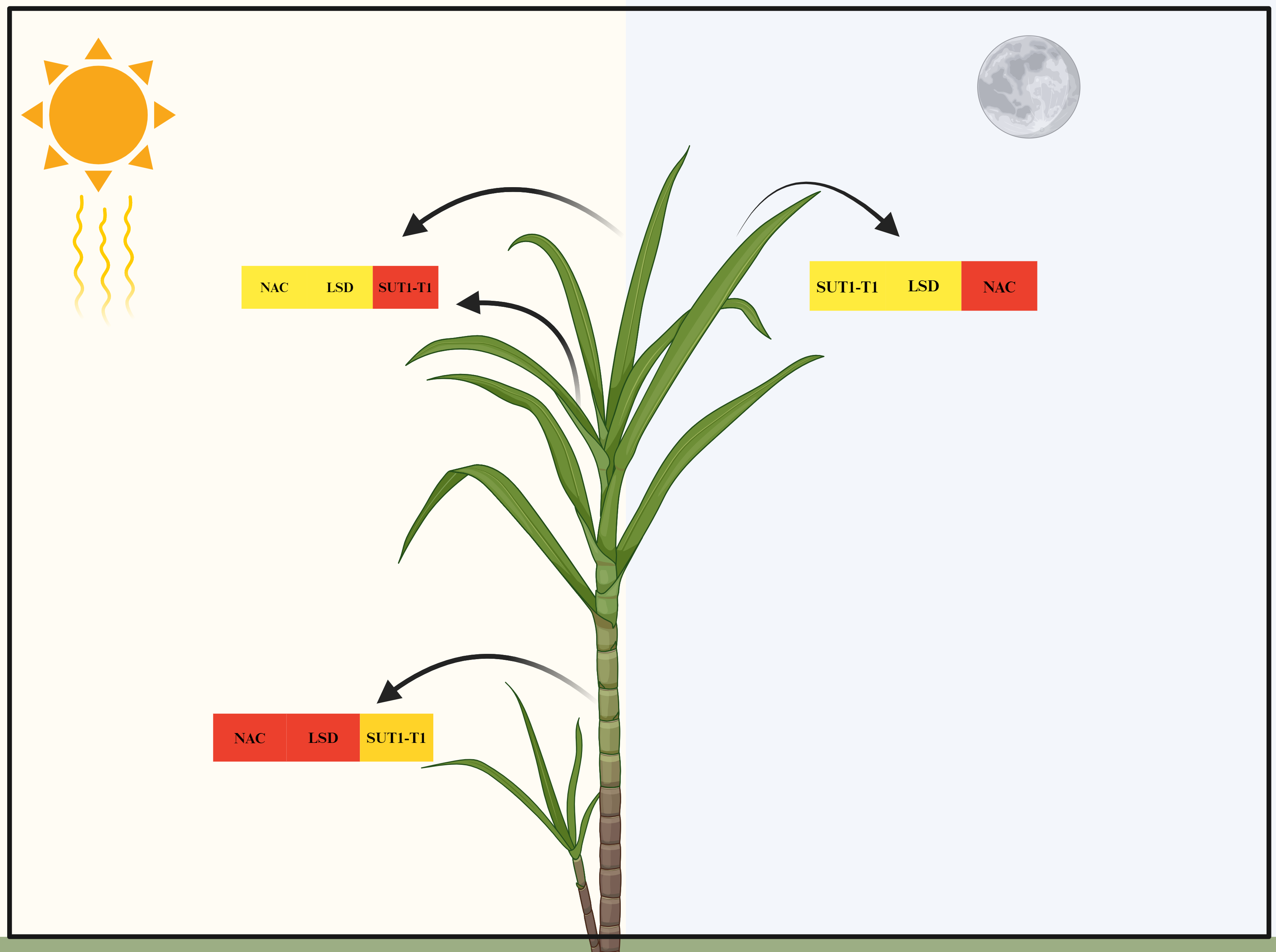

Supplement: Supplementary Figure S7 — Schematic representation of the expression pattern of LSD, NAC and SUT1-T1 at different stages of S. officinarum, during both daytime and night time. Red indicates higher expression levels, white and yellow indicates lower expression levels. [file Image7.png]
