# Supplementary material for: Genome-wide analysis of sugar transporter gene family in Erianthus rufipilus and Saccharum officinarum, expression profiling and identification of transcription factors
Source: Front Plant Sci. 2025 Jan 9;15:1502649. doi: 10.3389/fpls.2024.1502649 (PMC11755103; doi:10.3389/fpls.2024.1502649)
Supplement: Supplementary file 8 [file Table1.docx]

**Table S1** List of primer sequences used in the study.

| **qPCR primers** |  | **Gene Name** | **Seq (5'-3')** | **Tm** | **Product Size** |
| --- | --- | --- | --- | --- | --- |
|  | ***S. officinarum*** | S-INT3 F | GCTGCTTCTGGTCCTTACGT | 60 | 191 |
|  |  | S-INT3 R | AGGAGAAGAATTGGCCACCG | 60 |  |
|  |  | S-MST3-1 F | CTACACGCCCCAGATCCTTG | 60.18 | 162 |
|  |  | S-MST3-1 R | CGACGACCAGACACATCCAT | 59.83 |  |
|  |  | S-pGlcT1-T1 F | GGCACTTTGGGGACTTTGTG | 59.61 | 190 |
|  |  | S-pGlcT1-T1 R | CAACCTTAGCAAGCCAACGG | 59.76 |  |
|  |  | S-PLT11-T1 F | ACAGGTTAGTGACAGCGACG | 60.04 | 188 |
|  |  | S-PLT11-T1 R | CTTTGTTGGACGACATGCCG | 60.11 |  |
|  |  | S-SFP2-T2 F | GCTTGAAGGGTTTGGTGTCG | 59.69 | 185 |
|  |  | S-SFP2-T2 R | AGGCTCCGATCACTGCTAGA | 60.11 |  |
|  |  | S-STP4 F | GGTGGTGGTGGTGTTCATCT | 59.89 | 192 |
|  |  | S-STP4 R | TTGAAGTGGCAGAGCATGGT | 59.89 |  |
|  |  | S-SUT1-T1 F | GGCTATGCGGTCCTATTGCT | 59.96 | 165 |
|  |  | S-SUT1-T1 R | CAGCTCCGATGTCTGACGAA | 59.83 |  |
|  |  | S-VGT3 F | TTGGTCCTATTGGCTGGCTT | 59.3 | 171 |
|  |  | S-VGT3 R | CCCCGAATCCAGAGAAGAGC | 59.89 |  |
|  |  | S-SUT2-F | GGATACCACTGAGCACTGCA | 59.85 | 185 |
|  |  | S-SUT2-R | GCCAGGTCCAACATCCAGAA | 60 |  |
|  | ***E. rufipilus*** | E-INT1 F | TCCTTGCCTTGGCCTTCATA | 59 | 170 |
|  |  | E-INT1 R | GATTTCCGAGTTCACAGCCC | 60 |  |
|  |  | E-MST2 F | ATCTTCCCCACGCGTGTTAG | 60.11 | 166 |
|  |  | E-MST2 R | CGAAGATCAACGCCAAGCAG | 59.9 |  |
|  |  | E-pGlcT1 F | ATTGCTGCACTTTCCTTGGG | 59.03 | 197 |
|  |  | E-pGlcT1 R | TCAGAAGGTTCCCAAAGGCT | 59.08 |  |
|  |  | E-PLT15 F | ATGCCACCACTGACACTGAG | 59.96 | 161 |
|  |  | E-PLT15 R | CATCAGCGTCAGGTTGTAGC | 59.91 |  |
|  |  | E-SFP7 F | AAAGAAATACCGCATGCCCC | 59.89 | 162 |
|  |  | E-SFP7 R | AGAACCCCGATAGCTCCAAG | 59.88 |  |
|  |  | E-STP23 F | GCCTGTACACCTTCAGCTTG | 59.84 | 150 |
|  |  | E-STP23 R | TGAACACCTGTGCCTGTACA | 59.17 |  |
|  |  | E-SUT1 F | TTCGTTCATGTGGCTATGCG | 59.99 | 194 |
|  |  | E-SUT1 R | CCTTTGTGTCCCCTAGAGCA | 59.02 |  |
|  |  | E-VGT1 F | GGTTTCTGGTTGGCGTTACA | 58.69 | 172 |
|  |  | E-VGT1 R | CAATCGACATAAGCACCGGG | 59.06 |  |
|  |  | E-SUT2-F | GATGGGGCGTGAAGTTTACC | 59.91 | 176 |
|  |  | E-SUT2-R | CCCAAACCAATCTTGCACCA | 59.95 |  |
|  | **Y1H** | NAC-F | CCTCATCGGGCTCAAGAAGA | 59.17 | 179 |
|  |  | NAC-R | ACAGCACGATGTCCTCCTTG | 60.04 |  |
|  |  | LSD-F | GTTCCCCTTGCCCCGTATC | 60.15 | 199 |
|  |  | LSD-R | ATCCTCCGCAAACCAACTGT | 59.89 |  |
|  | **Reference genes** | Actin F | CTGCAATCTGGAACGGGAAGA | 60 | 161 |
|  |  | Actin R | TACACCTCATTTCCACCGCCA | 60.11 |  |
|  |  | eEF-F | CCTGCACTGTCATTGATGCT | 60.01 | 181 |
|  |  | eEF-R | CTGCCTGACACCAAGAGTGA | 60.21 |  |
| **Primers for Y1H assay** | **SUT1-T1** | SUT1-T1-Cis1-F | ATGAATTGAAAAGCTAGCCACCTCTTATTGTATCACTCTTTGT | 60 | 500 |
|  |  | SUT1-T1-Cis1-R | GAGCACATGCCTCGATCATCACAAAAATACTACATTGATAAATCATACAAATTTAAAACAACAAC | 60 |  |
|  |  | SUT1-T1-Cis2-F | ATGAATTGAAAAGCTTTGATGTGGAGCTCCGTTGTG | 60 | 500 |
|  |  | SUT1-T1-Cis2-R | GAGCACATGCCTCGATCCCCATCACATCAAAATGTTGGC | 60 |  |
|  |  | SUT1-T1-Cis3-F | ATGAATTGAAAAGCTATCTTCTTTTTACATAGTGGAATTTGTGGATGAATTTAGA | 60 | 500 |
|  |  | SUT1-T1-Cis3-R | GAGCACATGCCTCGAGCACAAACCACAGGCCTG | 60 |  |
|  |  | SUT1-T1-Cis4-F | ATGAATTGAAAAGCTGGCGTACGTGATTCGGTGAT | 60 | 500 |
|  |  | SUT1-T1-Cis4-R | GAGCACATGCCTCGATGAGGTGTGGGTGTGGAGC | 60 |  |
|  | **Sequencing primers** | pGADT7–F（T7) | TAATACGACTCACTATAGGGCGAGCGCCGCCATG | 56 | 1000 |
|  |  | pGADT7-R（ADR) | GTGAACTTGCGGGGTTTTTCAGTATCTACGATT | 56 |  |
