# Supplementary material for: Genome-wide analysis of sugar transporter gene family in Erianthus rufipilus and Saccharum officinarum, expression profiling and identification of transcription factors
Source: Front Plant Sci. 2025 Jan 9;15:1502649. doi: 10.3389/fpls.2024.1502649 (PMC11755103; doi:10.3389/fpls.2024.1502649)
Supplement: Supplementary file 9 [file Table2.docx]

| **Table S2a:** List of ST proteins with their gene ID, size in amino acids, molecular weight (KDa), sub cellular location and trans membrane domain (TMD) in *E. rufipilus*. | | | | | | | |  |
| --- | --- | --- | --- | --- | --- | --- | --- | --- |
|  | | **ST** | | | | | |  |
| **PROTEIN NAME** | | **ID** | **SIZE** | **PI** | **MW (KDa)** | **Location** | **TMD** |  |
|  |  |  |  |  |  |  |  |  |
| **MST** | MST1 | Erufi.04G037260 | 709 | 5.2 | 74.12 | plas | 10 |  |
|  | MST2 | Erufi.10G030260 | 761 | 4.74 | 80.91 | plas | 11 |  |
|  | MST3 | Erufi.01G032710 | 741 | 4.91 | 78.96 | vacu | 10 |  |
|  | MST4 | Erufi.04G011220 | 745 | 5.21 | 79.62 | plas | 11 |  |
|  | MST5 | Erufi.10G030660 | 745 | 5.57 | 79.92 | plas | 11 |  |
| **pGlcT** | pGlcT1 | Erufi.02G022210 | 547 | 6.21 | 57.97 | chlo | 9 |  |
|  | pGlcT2 | Erufi.04G013310 | 464 | 8.61 | 49.36 | plas | 10 |  |
| **PLT** | PLT1 | Erufi.05G025940 | 483 | 8.83 | 50.32 | vacu | 9 |  |
|  | PLT2 | Erufi.05G026140 | 479 | 8.79 | 50.22 | vacu | 12 |  |
|  | PLT3 | Erufi.08G012440 | 510 | 7.58 | 52.47 | cyto | 11 |  |
|  | PLT4 | Erufi.08G012480 | 502 | 7.57 | 52.06 | vacu | 11 |  |
|  | PLT5 | Erufi.06G016670 | 533 | 5.67 | 57.47 | plas | 9 |  |
|  | PLT6 | Erufi.05G025990 | 490 | 6.35 | 50.92 | vacu | 11 |  |
|  | PLT7 | Erufi.06G006370 | 518 | 9.38 | 54.76 | vacu | 12 |  |
|  | PLT8 | Erufi.08G012500 | 481 | 9.13 | 50.24 | plas | 12 |  |
|  | PLT9 | Erufi.08G012470 | 466 | 8.68 | 49.3 | chlo | 12 |  |
|  | PLT10 | Erufi.06G028800 | 542 | 6.18 | 57.29 | vacu | 10 |  |
|  | PLT11 | Erufi.06G028840 | 539 | 6.42 | 55.97 | plas | 11 |  |
|  | PLT12 | Erufi.01G048110 | 521 | 8.85 | 55.87 | plas | 11 |  |
|  | PLT13 | Erufi.09G009880 | 494 | 6.38 | 52.69 | plas | 10 |  |
|  | PLT14 | Erufi.01G048050 | 522 | 7.73 | 55.58 | plas | 12 |  |
|  | PLT15 | Erufi.01G033640 | 537 | 8.9 | 57.19 | plas | 10 |  |
|  | PLT16 | Erufi.02G037410 | 509 | 6.47 | 54.18 | vacu | 10 |  |
|  | PLT17 | Erufi.02G014500 | 501 | 8.98 | 52.38 | plas | 10 |  |
|  | PLT18 | Erufi.05G025960 | 500 | 9.24 | 52.26 | chlo | 11 |  |
|  | PLT19 | Erufi.05G025950 | 500 | 9.04 | 52.14 | chlo | 11 |  |
|  | PLT20 | Erufi.02G037330 | 510 | 8.93 | 54.24 | vacu | 12 |  |
|  | PLT21 | Erufi.02G037400 | 539 | 9.37 | 57.98 | plas | 11 |  |
|  | PLT22 | Erufi.02G037380 | 443 | 8.65 | 47.31 | cyto | 8 |  |
|  | PLT23 | Erufi.02G037360 | 348 | 8.77 | 37.14 | vacu | 6 |  |
|  | PLT24 | Erufi.02G037390 | 499 | 9.12 | 52.71 | vacu | 11 |  |
|  | PLT25 | Erufi.02G037350 | 510 | 9.04 | 53.85 | vacu | 10 |  |
| **SFP** | SFP1 | Erufi.09G024460 | 498 | 8.75 | 53.49 | plas | 12 |  |
|  | SFP2 | Erufi.09G025330 | 506 | 9.23 | 54.12 | plas | 12 |  |
|  | SFP3 | Erufi.01G037830 | 533 | 9.14 | 56.57 | chlo | 12 |  |
|  | SFP4 | Erufi.01G037820 | 496 | 6.25 | 52.69 | vacu | 12 |  |
|  | SFP5 | Erufi.03G011420 | 485 | 5.68 | 51.46 | plas | 12 |  |
|  | SFP6 | Erufi.09G024470 | 517 | 8.06 | 55.25 | plas | 11 |  |
|  | SFP7 | Erufi.09G024480 | 507 | 8.45 | 53.77 | plas | 10 |  |
|  | SFP8 | Erufi.09G024490 | 503 | 8.31 | 54.05 | plas | 12 |  |
| **STP** | STP1 | Erufi.02G000680 | 523 | 9.44 | 57.18 | plas | 11 |  |
|  | STP2 | Erufi.03G018930 | 704 | 8.83 | 77.81 | plas | 11 |  |
|  | STP3 | Erufi.02G035930 | 531 | 8.98 | 57.56 | vacu | 12 |  |
|  | STP4 | Erufi.06G011050 | 516 | 9.08 | 55.26 | vacu | 12 |  |
|  | STP5 | Erufi.01G031330 | 518 | 9.29 | 56.84 | vacu | 12 |  |
|  | STP6 | Erufi.01G055580 | 541 | 9.28 | 58.77 | plas | 12 |  |
|  | STP7 | Erufi.02G023120 | 518 | 9.3 | 55.72 | plas | 12 |  |
|  | STP8 | Erufi.02G010510 | 512 | 9.28 | 56.16 | plas | 12 |  |
|  | STP9 | Erufi.04G006230 | 521 | 8.99 | 57.05 | plas | 12 |  |
|  | STP10 | Erufi.01G020100 | 533 | 8.32 | 56.32 | vacu | 12 |  |
|  | STP11 | Erufi.05G022500 | 525 | 8.9 | 56.18 | vacu | 12 |  |
|  | STP12 | Erufi.05G022510 | 516 | 9.12 | 55.46 | vacu | 12 |  |
|  | STP13 | Erufi.05G022680 | 454 | 7.58 | 49.003 | cyto | 12 |  |
|  | STP14 | Erufi.07G008530 | 514 | 7.56 | 55.58 | plas | 12 |  |
|  | STP15 | Erufi.01G046730 | 517 | 8.93 | 57.001 | plas | 12 |  |
|  | STP16 | Erufi.02G019900 | 518 | 9.22 | 56.71 | plas | 12 |  |
|  | STP17 | Erufi.10G004340 | 525 | 9.91 | 55.27 | vacu | 12 |  |
|  | STP18 | Erufi.09G016250 | 509 | 9.63 | 54.54 | plas | 12 |  |
|  | STP19 | Erufi.04G019600 | 523 | 8.99 | 56.54 | vacu | 12 |  |
|  | STP20 | Erufi.06G011060 | 509 | 9.58 | 54.29 | vacu | 12 |  |
|  | STP21 | Erufi.06G000630 | 506 | 9.12 | 55.46 | vacu | 12 |  |
|  | STP22 | Erufi.06G010990 | 510 | 9.47 | 54.38 | vacu | 12 |  |
|  | STP23 | Erufi.06G011090 | 512 | 9.58 | 54.15 | vacu | 12 |  |
|  | STP24 | Erufi.06G011040 | 509 | 9.34 | 55.14 | plas | 12 |  |
|  | STP25 | Erufi.06G011030 | 502 | 9.78 | 54.49 | vacu | 12 |  |
|  | STP26 | Erufi.04G034510 | 526 | 9.01 | 56.11 | plas | 12 |  |
|  | STP27 | Erufi.09G014150 | 369 | 10 | 41.69 | cyto | 8 |  |
|  | SUT1 | Erufi.01G049920 | 521 | 8.67 | 55.09 | plas | 12 |  |
| **SUT** | SUT2 | Erufi.04G036740 | 595 | 5.95 | 63.26 | plas | 12 |  |
|  | SUT3 | Erufi.01G026000 | 508 | 7.45 | 53.56 | plas | 12 |  |
|  | SUT4 | Erufi.04G019750 | 534 | 8.61 | 56.56 | plas | 12 |  |
|  | SUT5 | Erufi.07G025730 | 523 | 8.53 | 55.23 | plas | 12 |  |
| **VGT** | VGT1 | Erufi.01G029880 | 511 | 5.31 | 54.72 | plas | 12 |  |
|  | VGT2 | Erufi.01G003560 | 561 | 9.73 | 58.91 | chlo | 12 |  |
| **INT** | INT1 | Erufi.06G013950 | 506 | 5.66 | 53.79 | plas | 12 |  |
|  | INT2 | Erufi.02G005420 | 590 | 8.68 | 63.2 | cyto | 12 |  |
|  | INT3 | Erufi.06G015380 | 586 | 8.83 | 63.34 | plas | 12 |  |
|  | INT4 | Erufi.02G015680 | 573 | 8.56 | 61.196 | vacu | 12 |  |

| **Table S2b:** List of ST proteins with their gene ID, size in amino acids, molecular weight (KDa), sub cellular location and trans membrane domain (TMD) in *S. officinarum*. | | | | | | | |  |
| --- | --- | --- | --- | --- | --- | --- | --- | --- |
|  | | **ST** | | | | | |  |
| **PROTEIN NAME** | | **ID** | **SIZE** | **PI** | **MW (KDa)** | **Location** | **TMD** |  |
|  |  |  |  |  |  |  |  |  |
| **MST** | MST1-1 | Soffic.03G0039330-1A | 700 | 5.09 | 73.383 | plas | 10 |  |
|  | MST1-2 | Soffic.04G0032640-1P | 658 | 5.08 | 68.665 | plas | 8 |  |
|  | MST1-T1 | LAp.03E0040640 | 700 | 5.09 | 73.383 | plas | 10 |  |
|  | MST2-1 | Soffic.06G0004520-3E | 760 | 4.76 | 80.687 | plas | 11 |  |
|  | MST2-2 | LAp.10F0024120 | 760 | 4.76 | 80.687 | plas | 11 |  |
|  | MST3-1 | Soffic.01G0031070-3D | 741 | 4.88 | 79.069 | vacu | 10 |  |
|  | MST4 | Soffic.04G0008940-6H | 745 | 5.16 | 79.635 | plas | 11 |  |
| **pGlcT** | pGlcT1 | Soffic.02G0016850-5G | 550 | 6.42 | 58.367 | chlo | 9 |  |
|  | pGlcT1-T1 | Soffic.02G0018140-1A | 572 | 6.68 | 61.041 | chlo | 9 |  |
|  | pGlcT2 | Soffic.04G0011830-1A | 487 | 8.64 | 52.425 | chlo | 10 |  |
| **PLT** | PLT1 | Soffic.05G0018470-1PE | 399 | 8.15 | 41.366 | vacu | 9 |  |
|  | PLT3-1 | Soffic.08G0010460-3D | 501 | 7.61 | 52.359 | cyto | 11 |  |
|  | PLT3-2 | LAp.08B0008540 | 516 | 8.19 | 52.803 | cyto | 11 |  |
|  | PLT4 | Soffic.06G0014460-6F | 533 | 5.55 | 57.481 | plas | 9 |  |
|  | PLT5 | LAp.05B0018240 | 434 | 7.61 | 45.216 | cyto | 8 |  |
|  | PLT6-1 | Soffic.06G0004840-2D | 519 | 9.39 | 54.874 | vacu | 12 |  |
|  | PLT6-2 | Soffic.06G0006330-1A | 519 | 9.39 | 54.892 | vacu | 12 |  |
|  | PLT7 | Soffic.08G0010080-3P | 480 | 9.13 | 50.094 | plas | 12 |  |
|  | PLT7-T1 | Soffic.08G0010060-3C | 488 | 8.51 | 51.464 | cyto | 12 |  |
|  | PLT8-1 | Soffic.01G0029060-4E | 486 | 8.84 | 50.933 | cyto | 10 |  |
|  | PLT8-2 | Soffic.05G0012100-2C | 509 | 9.11 | 52.972 | cyto | 11 |  |
|  | PLT9 | Soffic.06G0024170-5E | 540 | 7.53 | 57.106 | vacu | 10 |  |
|  | PLT10 | LAp.01F0048780 | 463 | 6.02 | 48.208 | plas | 10 |  |
|  | PLT11 | LAp.01E0047760 | 521 | 8.85 | 55.816 | plas | 11 |  |
|  | PLT11-T1 | LAp.01C0047490 | 521 | 8.85 | 55.816 | plas | 11 |  |
|  | PLT12 | LAp.01F0042400 | 526 | 8.6 | 55.979 | plas | 10 |  |
|  | PLT12-T1 | Soffic.01G0046740-1A | 523 | 7.72 | 55.711 | plas | 12 |  |
|  | PLT12-T2 | LAp.01C0047450 | 523 | 7.72 | 55.745 | plas | 12 |  |
|  | PLT13-T1 | Soffic.01G0030860-1P | 537 | 8.89 | 57.224 | plas | 10 |  |
|  | PLT14 | Soffic.02G0031300-6G | 509 | 6.47 | 54.112 | vacu | 10 |  |
|  | PLT15 | Soffic.02G0007690-2B | 501 | 9.05 | 52.479 | vacu | 10 |  |
|  | PLT17 | Soffic.02G0031270-7G | 512 | 8.57 | 54.547 | vacu | 12 |  |
|  | PLT17-T1 | Soffic.02G0031270-3P | 504 | 9.04 | 53.857 | plas | 11 |  |
|  | PLT18 | Soffic.02G0013030-2B | 510 | 9.04 | 53.958 | vacu | 10 |  |
|  | PLT18_T1 | Soffic.02G0032210-3C | 527 | 9.34 | 55.618 | vacu | 10 |  |
|  | PLT18-T2 | Soffic.02G0013040-2B | 463 | 8.99 | 59.74 | vacu | 7 |  |
| **SFP** | SFP2 | LAp.01G0016880 | 432 | 9.23 | 46.115 | plas | 10 |  |
|  | SFP2-T2 | Soffic.09G0023240-4F | 506 | 9.3 | 54.164 | plas | 12 |  |
|  | SFP4 | LAp.01H0033380 | 417 | 10.03 | 44.688 | chlo | 10 |  |
|  | SFP6 | Soffic.03G0009950-3D | 436 | 5.1 | 47.279 | plas | 7 |  |
|  | SFP7 | Soffic.09G0019430-6P | 310 | 5.7 | 33.109 | vacu | 6 |  |
|  | SFP8-T1 | Soffic.09G0022150-1A | 354 | 7.58 | 39.072 | vacu | 7 |  |
|  | SFP4-T1 | Soffic.01G0026950-7G | 397 | 5.33 | 41.991 | vacu | 8 |  |
|  | SFP5 | LAp.03E0008910 | 436 | 5.1 | 47.279 | plas | 7 |  |
| **STP** | STP1 | Soffic.02G0001250-1P | 523 | 9.43 | 57.163 | plas | 11 |  |
|  | STP2 | Soffic.09G0015080-6F | 510 | 9.65 | 54.638 | plas | 9 |  |
|  | STP3 | Soffic.03G0016930-6F | 509 | 9.46 | 55.641 | vacu | 12 |  |
|  | STP4 | LAp.02E0029530 | 531 | 8.98 | 57.596 | plas | 11 |  |
|  | STP5 | Soffic.06G0008440-2PH | 516 | 9.09 | 55.202 | vacu | 12 |  |
|  | STP6 | LAp.01E0033580 | 518 | 9.2 | 56.648 | plas | 11 |  |
|  | STP6-T1 | Soffic.01G0008870-1A | 541 | 9.28 | 58.721 | plas | 11 |  |
|  | STP6-T2 | Soffic.01G0055320-1A | 541 | 9.28 | 58.735 | plas | 11 |  |
|  | STP8-1 | Soffic.02G0000200-2B | 512 | 9.22 | 56.07 | plas | 9 |  |
|  | STP8-2 | Soffic.02G0012310-1A | 512 | 9.13 | 56.057 | plas | 9 |  |
|  | STP9 | Soffic.04G0003920-2B | 521 | 8.99 | 57.057 | plas | 11 |  |
|  | STP10 | Soffic.01G0055830-1A | 533 | 8.91 | 56.541 | vacu | 11 |  |
|  | STP10-T1 | Soffic.01G0017350-2B | 533 | 9.01 | 56.544 | vacu | 11 |  |
|  | STP11-T1 | LAp.05G0016620 | 516 | 9.35 | 55.336 | vacu | 12 |  |
|  | STP12 | LAp.07G0005260 | 514 | 7.56 | 55.614 | plas | 11 |  |
|  | STP13 | LAp.01E0046310 | 517 | 8.93 | 56.941 | plas | 11 |  |
|  | STP14 | Soffic.02G0015560-1A | 518 | 9.14 | 56.673 | plas | 10 |  |
|  | STP16 | LAp.10F0001760 | 524 | 9.79 | 55.068 | vacu | 12 |  |
|  | STP17 | Soffic.09G0014730-3C | 509 | 9.72 | 54.549 | plas | 9 |  |
|  | STP18 | Soffic.06G0009980-1P | 516 | 9.2 | 55.164 | vacu | 12 |  |
|  | STP19 | Soffic.04G0016430-4E | 523 | 9.25 | 56.637 | vacu | 10 |  |
|  | STP20 | Soffic.06G0009590-6F | 510 | 9.58 | 54.425 | vacu | 10 |  |
|  | STP20-T1 | Soffic.06G0009830-5E | 510 | 9.58 | 54.409 | vacu | 10 |  |
|  | STP25-T1 | Soffic.06G0009850-5E | 512 | 9.58 | 54.113 | vacu | 10 |  |
|  | STP28-T1 | Soffic.06G0009910-7G | 508 | 9.71 | 54.489 | vacu | 11 |  |
|  | STP29 | Soffic.04G0032120-5G | 526 | 9.2 | 56.042 | plas | 11 |  |
|  | STP29-T1 | Soffic.04G0030630-1A | 526 | 9.2 | 56.012 | plas | 11 |  |
| **SUT** | SUT1-T1 | LAp.01E0049080 | 521 | 8.79 | 55.052 | plas | 10 |  |
|  | SUT2-1 | Soffic.04G0007330-2E | 595 | 6.06 | 63.209 | plas | 11 |  |
|  | SUT2-2 | Soffic.04G0033650-1A | 595 | 5.73 | 63.014 | plas | 11 |  |
|  | SUT3 | LAp.01B0023620 | 508 | 7.46 | 53.459 | plas | 10 |  |
|  | SUT4 | LAp.08D0018580 | 501 | 8.6 | 53.461 | chlo | 10 |  |
|  | SUT5 | Soffic.04G0018630-5P | 534 | 8.61 | 56.401 | plas | 12 |  |
|  | SUT6 | Soffic.07G0019280-4E | 529 | 8.47 | 55.723 | plas | 10 |  |
| **VGT** | VGT1 | Soffic.01G0039230-1P | 537 | 5.83 | 57.838 | plas | 12 |  |
|  | VGT2 | Soffic.01G0036600-5G | 512 | 5.2 | 54.794 | plas | 11 |  |
|  | VGT3 | Soffic.01G0003530-3F | 561 | 9.73 | 58.982 | chlo | 11 |  |
|  | VGT3-T1 | LAp.01H0019910 | 561 | 9.73 | 58.982 | chlo | 11 |  |
| **INT** | INT1 | Soffic.06G0011760-2B | 506 | 5.44 | 53.798 | plas | 12 |  |
|  | INT2 | Soffic.02G0016610-4D | 586 | 8.69 | 62.591 | cyto | 12 |  |
|  | INT3 | LAp.06G0013710 | 586 | 8.83 | 63.358 | plas | 12 |  |
|  | INT4 | LAp.02F0008700 | 573 | 8.56 | 61.168 | vacu | 12 |  |
