# Supplementary material for: Genome-wide analysis of sugar transporter gene family in Erianthus rufipilus and Saccharum officinarum, expression profiling and identification of transcription factors
Source: Front Plant Sci. 2025 Jan 9;15:1502649. doi: 10.3389/fpls.2024.1502649 (PMC11755103; doi:10.3389/fpls.2024.1502649)
Supplement: Supplementary file 10 [file Table3.docx]

| **WGD or Segmental** | | **Dispersed** |
| --- | --- | --- |
| LAp.02F0008700 (INT4) | LAp.03E0008910 (SFP5) | Soffic.02G0016610-4D (INT2) |
| LAp.06G0013710 (INT3) | Soffic.09G0023240-4F (SFP2-T2) | Soffic.03G0039330-1A (MST1-1) |
| Soffic.06G0011760-2B (INT1) | LAp.01E0049080 (SUT1-T1) | Soffic.06G0004520-3E (MST2-1) |
| Soffic.01G0031070-3D (MST3-1) | Soffic.04G0018630-5P (SUT5) | LAp.03E0040640 (MST1-T1) |
| Soffic.04G0008940-6H (MST4) | Soffic.07G0019280-4E (SUT6) | LAp.01C0047490 (PLT11-T1) |
| Soffic.04G0032640-1P (MST1-2) | Soffic.04G0033650-1A (SUT2-2) | LAp.01E0047760 (PLT11) |
| LAp.10F0024120 (MST2-2) | Soffic.01G0055830-1A (STP10) | LAp.01C0047450 (PLT12-T2) |
| Soffic.02G0018140-1A (pGlcT1-T1) | Soffic.01G0017350-2B (STP10-T1) | Soffic.08G0010080-3P (PLT7) |
| Soffic.02G0016850-5G (pGlcT1) | Soffic.01G0008870-1A (STP6-T1) | Soffic.02G0031300-6G (PLT14) |
| Soffic.04G0011830-1A (pGlcT2) | LAp.01E0033580 (STP6) | Soffic.02G0031270-3P (PLT17-T1) |
| Soffic.01G0030860-1P (PLT13-T1) | LAp.01E0046310 (STP13) | Soffic.02G0013040-2B (PLT18-T2) |
| LAp.01F0042400 (PLT12) | Soffic.02G0015560-1A (STP14) | Soffic.01G0029060-4E (PLT8-1) |
| Soffic.01G0046740-1A (PLT12-T1) | LAp.02E0029530 (STP4) | Soffic.05G0012100-2C (PLT8-2) |
| Soffic.02G0007690-2B (PLT15) | Soffic.03G0016930-6F (STP3) | LAp.01H0033380 (SFP4) |
| Soffic.08G0010460-3D (PLT3-1) | Soffic.04G0016430-4E (STP19) | Soffic.09G0022150-1A (SFP8-T1) |
| Soffic.08G0010060-3C (PLT7-T1) | Soffic.04G0003920-2B (STP9) | Soffic.09G0019430-6P (SFP7) |
| Soffic.02G0031270-7G (PLT17) | Soffic.04G0030630-1A (STP29-T1) | LAp.01G0016880 (SFP2) |
| Soffic.02G0032210-3C (PLT18-T1) | Soffic.04G0032120-5G (STP29) | LAp.01B0023620 (SUT3) |
| Soffic.02G0013030-2B (PLT18) | Soffic.06G0009830-5E (STP20-T1) | LAp.08D0018580 (SUT4) |
| Soffic.06G0024170-5E (PLT9) | Soffic.06G0009590-6F (STP20) | Soffic.04G0007330-2E (SUT2-1) |
| Soffic.06G0014460-6F (PLT4) | LAp.05G0016620 (STP11-T1) | LAp.01H0019910 (VGT3-T1) |
| LAp.01F0048780 (PLT10) | LAp.07G0005260 (STP12) | Soffic.02G0001250-1P (STP1) |
| Soffic.06G0004840-2D (PLT6-1) | Soffic.09G0014730-3C (STP17) | Soffic.02G0000200-2B (STP8-1) |
| LAp.05B0018240 (PLT5) | Soffic.09G0015080-6F (STP2) | Soffic.06G0009850-5E (STP25-T1) |
| Soffic.06G0006330-1A (PLT6-2) | LAp.10F0001760 (STP16) | Soffic.06G0009980-1P (STP18) |
| LAp.08B0008540 (PLT3-2) | Soffic.02G0012310-1A (STP8-2) | Soffic.06G0008440-2PH (STP5) |
| Soffic.05G0018470-1PE (PLT1) | Soffic.01G0055320-1A (STP6-T2) | Soffic.06G0009910-7G (STP28-T1) |
| Soffic.03G0009950-3D (SFP6) | Soffic.01G0039230-1P (VGT1) | Soffic.01G0003530-3F (VGT3) |
| Soffic.01G0026950-7G (SFP4-T1) | Soffic.01G0036600-5G (VGT2) | Soffic.01G0036600-5G (VGT2) |

**Table S3a** List of ST genes undergoing WGD or segmental and dispersed evolution in *S. officinarum.*

**Table S3b** List of ST genes undergoing WGD or segmental and dispersed evolution in *E. rufipilus.*

| **WGD or Segmental** | | **Dispersed** |
| --- | --- | --- |
| Erufi.06G013950 (INT1) | Erufi.03G018930 (STP2) | Erufi.04G037260 (MST1) |
| Erufi.02G005420 (INT2) | Erufi.02G035930 (STP3) | Erufi.10G030660 (MST5) |
| Erufi.06G015380 (INT3) | Erufi.06G011050 (STP4) | Erufi.08G012480 (PLT4) |
| Erufi.02G015680 (INT4) | Erufi.01G055580 (STP6) | Erufi.05G025990 (PLT6) |
| Erufi.10G030260 (MST2) | Erufi.02G023120 (STP7) | Erufi.08G012500 (PLT8) |
| Erufi.01G032710 (MST3) | Erufi.02G010510 (STP8) | Erufi.06G028840 (PLT11) |
| Erufi.04G011220 (MST4) | Erufi.04G006230 (STP9) | Erufi.09G009880 (PLT13) |
| Erufi.02G022210 (pGlcT1) | Erufi.01G020100 (STP10) | Erufi.02G037410 (PLT16) |
| Erufi.04G013310 (pGlcT2) | Erufi.07G008530 (STP14) | Erufi.05G025960 (PLT18) |
| Erufi.05G025940 (PLT1) | Erufi.02G019900 (STP16) | Erufi.02G037400 (PLT21) |
| Erufi.05G026140 (PLT2) | Erufi.10G004340 (STP17) | Erufi.02G037380 (PLT22) |
| Erufi.08G012440 (PLT3) | Erufi.09G016250 (STP18) | Erufi.02G037360 (PLT23) |
| Erufi.06G016670 (PLT5) | Erufi.04G019600 (STP19) | Erufi.02G037390 (PLT24) |
| Erufi.06G006370 (PLT7) | Erufi.06G011060 (STP20) | Erufi.02G037350 (PLT25) |
| Erufi.08G012470 (PLT9) | Erufi.06G000630 (STP21) | Erufi.01G037830 (SFP3) |
| Erufi.06G028800 (PLT10) | Erufi.06G010990 (STP22) | Erufi.09G024470 (SFP6) |
| Erufi.01G048110 (PLT12) | Erufi.06G011090 (STP23 | Erufi.09G024480 (SFP7) |
| Erufi.01G048050 (PLT14) | Erufi.06G011030 (STP25) | Erufi.09G024490 (SFP8) |
| Erufi.01G033640 (PLT15) | Erufi.04G034510 (STP26) | Erufi.02G000680 (STP1) |
| Erufi.02G014500 (PLT17) | Erufi.09G014150 (STP27) | Erufi.01G031330 (STP5) |
| Erufi.05G025950 (PLT19) | Erufi.01G049920 (SUT1) | Erufi.05G022500 (STP11) |
| Erufi.02G037330 (PLT20) | Erufi.01G026000 (SUT3) | Erufi.05G022510 (STP12) |
| Erufi.09G024460 (SFP1) | Erufi.04G019750 (SUT4) | Erufi.05G022680 (STP13) |
| Erufi.09G025330 (SFP2) | Erufi.07G025730 (SUT5) | Erufi.01G046730 (STP15) |
| Erufi.01G037820 (SFP4) | Erufi.01G029880 (VGT1) | Erufi.06G011040 (STP24) |
| Erufi.03G011420 (SFP5) | Erufi.01G003560 (VGT2) | Erufi.04G036740 (SUT2) |
