# Supplementary material for: Genome-wide analysis of sugar transporter gene family in Erianthus rufipilus and Saccharum officinarum, expression profiling and identification of transcription factors
Source: Front Plant Sci. 2025 Jan 9;15:1502649. doi: 10.3389/fpls.2024.1502649 (PMC11755103; doi:10.3389/fpls.2024.1502649)
Supplement: Supplementary file 11 [file Table4.docx]

| **Gene Pairs** | | **Ka** | **Ks** | **Ka_Ks** | **Time (MYA)** |
| --- | --- | --- | --- | --- | --- |
| Soffic.02G0032210-3C (PLT18-T1) | Soffic.02G0013040-2B (PLT18-T2) | 0.1229683 | 0.2057885 | 0.597546934 | 15.82988418 |
| Soffic.01G0030860-1P (PLT13-T1) | Soffic.01G0033030-2P (PLT13) | 0.0050619 | 0.0023753 | 2.131052427 | 0.1827153 |
| Soffic.08G0010060-3C (PLT7-T1) | Soffic.08G0010080-3P (PLT7) | 0.0829659 | 0.3311611 | 0.2505302 | 25.47393091 |
| Soffic.01G0029060-4E (PLT8-1) | Soffic.05G0012100-2C (PLT8-2) | 0.0057739 | 0.0220154 | 0.262265276 | 1.693495481 |
| Soffic.06G0006330-1A (PLT6-2) | Soffic.06G0004840-2D (PLT6-1) | 0.0008829 | 0.0118956 | 0.074218582 | 0.915043888 |
| Soffic.06G0024170-5E (PLT9) | LAp.01F0048780 (PLT10) | 0.1643491 | 0.4572874 | 0.359400006 | 35.17595499 |
| Soffic.08G0010460-3D (PLT3-1) | LAp.08B0008540 (PLT3-2) | 0.0036549 | 0.0187108 | 0.195337853 | 1.439290273 |
| LAp.02F0008700 (INT4) | LAp.06G0013710 (INT3) | 0.1977674 | 1.1389108 | 0.173646057 | 87.60852617 |
| Soffic.02G0018140-1A (pGlcT1-T1) | Soffic.02G0016850-5G (pGlcT1) | 0.0008186 | 0.0047244 | 0.173260017 | 0.363417313 |
| Soffic.01G0031070-3D (MST3-1) | Soffic.04G0008940-6H (MST4) | 0.157339 | 1.4731539 | 0.106804186 | 113.3195327 |
| LAp.01H0033380 (SFP4) | Soffic.01G0026950-7G (SFP4-T1) | 0.1783135 | 0.8685687 | 0.205295747 | 66.81297828 |
| Soffic.09G0022150-1A (SFP8-T1) | Soffic.09G0019430-6P (SFP7) | 0.0353402 | 0.0674028 | 0.524313863 | 5.184834029 |
| LAp.01G0016880 (SFP2) | Soffic.09G0023240-4F (SFP2-T2) | 0.003131 | 0.0089866 | 0.348404756 | 0.691279057 |
| Soffic.06G0009980-1P (STP18) | Soffic.06G0008440-2PH (STP5) | 0.0061779 | 0.0122878 | 0.502764267 | 0.945215617 |
| Soffic.06G0009590-6F (STP20) | Soffic.06G0009830-5E (STP20-T1) | 0.0017804 | 0.0074381 | 0.239365114 | 0.572159807 |
| Soffic.09G0014730-3C (STP17) | Soffic.09G0015080-6F (STP2) | 0.0066826 | 0.0319469 | 0.209176767 | 2.457454453 |
| Soffic.01G0008870-1A (STP6-T1) | Soffic.01G0055320-1A (STP6-T2) | 0.0008293 | 0.0024048 | 0.344851156 | 0.184985514 |
| Soffic.02G0000200-2B (STP8-1) | Soffic.02G0012310-1A (STP8-2) | 0.0026354 | 0.0550465 | 0.047876488 | 4.234348578 |
| Soffic.04G0003920-2B (STP9) | LAp.07G0005260 (STP12) | 0.3505054 | 0.5851918 | 0.598958145 | 45.01475239 |
| Soffic.02G0001250-1P (STP1) | LAp.02E0029530 (STP4) | 0.1486521 | 0.3550185 | 0.418716552 | 27.30911885 |
| Soffic.04G0030630-1A (STP29-T1) | Soffic.04G0032120-5G (STP29) | 0.0008626 | 0.0218462 | 0.039483508 | 1.680477284 |
| Soffic.04G0018630-5P (SUT5) | Soffic.07G0019280-4E (SUT6) | 0.0435042 | 0.1150446 | 0.378150744 | 8.849585084 |
| LAp.01B0023620 (SUT3) | LAp.01E0049080 (SUT1-T1) | 0.2135196 | 1.175782 | 0.181597943 | 90.44477293 |
| Soffic.04G0007330-2E (SUT2-1) | Soffic.04G0033650-1A (SUT2-2) | 0.0060128 | 0.0411846 | 0.145996639 | 3.168044146 |

**Table S4a** Ratio of synonymous and non-synonymous substitution rate in ST gene families in *S. officinarum.*

| **Gene Pairs** | | **Ka** | **Ks** | **Ka_Ks** | **Time (MYA)** |
| --- | --- | --- | --- | --- | --- |
| E.rufi.02G037360 (PLT23) | E.rufi.02G037350 (PLT25) | 0.053422 | 0.107396 | 0.497432 | 8.261266741 |
| E.rufi.09G009880 (PLT13) | E.rufi.01G048050 (PLT14) | 0.025691 | 0.109413 | 0.234811 | 8.416422611 |
| E.rufi.08G012440 (PLT3) | E.rufi.08G012480 (PLT4) | 0.060492 | 0.155903 | 0.388012 | 11.99254398 |
| E.rufi.08G012500 (PLT8) | E.rufi.08G012470 (PLT9) | 0.078487 | 0.368172 | 0.21318 | 28.32090285 |
| E.rufi.05G025940 (PLT1) | E.rufi.05G026140 (PLT2) | 0.146679 | 0.408802 | 0.358802 | 31.44631664 |
| E.rufi.02G014500 (PLT17) | E.rufi.05G025950 (PLT19) | 0.050752 | 0.162985 | 0.311392 | 12.53730158 |
| E.rufi.06G028800 (PLT10) | E.rufi.06G028840 (PLT11) | 0.165107 | 0.477567 | 0.345725 | 36.73592634 |
| E.rufi.01G029880 (VGT1) | E.rufi.01G003560 (VGT2) | 0.333801 | 3.384114 | 0.098638 | 260.3164325 |
| E.rufi.04G011220 (MST4) | E.rufi.10G030660 (MST5) | 0.077451 | 0.546044 | 0.14184 | 42.00339514 |
| E.rufi.06G015380 (INT3) | E.rufi.02G015680 (INT4) | 0.200354 | 1.154505 | 0.173541 | 88.80806293 |
| E.rufi.02G022210 (pGlcT1) | E.rufi.04G013310 (pGlcT2) | 0.561088 | 2.628451 | 0.213467 | 202.1885005 |
| E.rufi.01G037830 (SFP3) | E.rufi.01G037820 (SFP4) | 0.151838 | 0.845271 | 0.179632 | 65.02087497 |
| E.rufi.09G024470 (SFP6) | E.rufi.09G024480 (SFP7) | 0.071186 | 0.089369 | 0.79654 | 6.874551574 |
| E.rufi.06G010990 (STP22) | E.rufi.06G011090 (STP23) | 0.216163 | 0.52498 | 0.411755 | 40.38306609 |
| E.rufi.05G022500 (STP11) | E.rufi.05G022680 (STP13) | 0.03592 | 0.086241 | 0.416506 | 6.633922705 |
| E.rufi.10G004340 (STP17) | E.rufi.09G016250 (STP18) | 0.197537 | 0.591139 | 0.334164 | 45.47219609 |
| E.rufi.01G031330 (STP5) | E.rufi.01G055580 (STP6) | 0.136602 | 1.163726 | 0.117383 | 89.51741174 |
| E.rufi.02G023120 (STP7) | E.rufi.02G019900 (STP16) | 0.34429 | 0.411517 | 0.836638 | 31.65512536 |
| E.rufi.02G000680 (STP1) | E.rufi.02G035930 (STP3) | 0.152745 | 0.319794 | 0.477635 | 24.5995747 |
| E.rufi.04G006230 (STP9) | E.rufi.07G008530 (STP14) | 0.354377 | 0.598227 | 0.592379 | 46.0174433 |
| E.rufi.04G019750 (SUT4) | E.rufi.07G025730 (SUT5) | 0.039843 | 0.092193 | 0.432166 | 7.091794336 |
| E.rufi.01G049920 (SUT1) | E.rufi.01G026000 (SUT3) | 0.227927 | 1.092833 | 0.208565 | 84.0640673 |
| E.rufi.01G033640 (PLT15) | E.rufi.02G037330 (PLT20) | 0.138139 | 0.448967 | 0.307682 | 34.53590548 |
| E.rufi.04G037260 (MST1) | E.rufi.10G030260 (MST2) | 0.18547 | 0.591723 | 0.31344 | 45.51713593 |
| E.rufi.01G020100 (STP10) | E.rufi.04G034510 (STP26) | 0.223308 | 0.601697 | 0.37113 | 46.28440025 |

**Table S4b** Ratio of synonymous and non-synonymous substitution rate in ST gene families in *E. rufipilum.*
